# A novel method for targeting lymphatic vessel imaging: methylene blue nanoparticle integrated with dissolvable microneedles

**DOI:** 10.1093/burnst/tkaf067

**Published:** 2026-01-28

**Authors:** Chengyao Han, Beibei Wu, Chunxiao Cui, Peiru Min, Xinxian Meng, Yuhao Sun, Ke Wen, Chuanliang Feng, Yixin Zhang, Xueqian Wang, Ke Li

**Affiliations:** Department of Plastic and Reconstructive Surgery, Shanghai Ninth People’s Hospital, Shanghai Jiao Tong University School of Medicine, No. 639, Zhizaoju Road, Huangpu District, Shanghai 200011, China; State Key Lab of Metal Matrix Composites, Shanghai Key Laboratory for Molecular Engineering of Chiral Drugs, School of Materials Science and Engineering, Shanghai Jiao Tong University, No. 800, Dongchuan Road, Minhang District, Shanghai 200240, China; Department of Burns and Plastic Surgery, Shanghai Children’s Medical Center, Shanghai Jiao Tong University, No. 1678, Dongfang Road, Pudong New District, Shanghai 200127, China; Department of Plastic and Reconstructive Surgery, Shanghai Ninth People’s Hospital, Shanghai Jiao Tong University School of Medicine, No. 639, Zhizaoju Road, Huangpu District, Shanghai 200011, China; Department of Plastic and Reconstructive Surgery, Shanghai Ninth People’s Hospital, Shanghai Jiao Tong University School of Medicine, No. 639, Zhizaoju Road, Huangpu District, Shanghai 200011, China; Department of Plastic and Reconstructive Surgery, Shanghai Ninth People’s Hospital, Shanghai Jiao Tong University School of Medicine, No. 639, Zhizaoju Road, Huangpu District, Shanghai 200011, China; Department of Plastic and Reconstructive Surgery, Shanghai Ninth People’s Hospital, Shanghai Jiao Tong University School of Medicine, No. 639, Zhizaoju Road, Huangpu District, Shanghai 200011, China; State Key Lab of Metal Matrix Composites, Shanghai Key Laboratory for Molecular Engineering of Chiral Drugs, School of Materials Science and Engineering, Shanghai Jiao Tong University, No. 800, Dongchuan Road, Minhang District, Shanghai 200240, China; Department of Plastic and Reconstructive Surgery, Shanghai Ninth People’s Hospital, Shanghai Jiao Tong University School of Medicine, No. 639, Zhizaoju Road, Huangpu District, Shanghai 200011, China; Department of Plastic and Reconstructive Surgery, Shanghai Ninth People’s Hospital, Shanghai Jiao Tong University School of Medicine, No. 639, Zhizaoju Road, Huangpu District, Shanghai 200011, China; Department of Plastic and Reconstructive Surgery, Shanghai Ninth People’s Hospital, Shanghai Jiao Tong University School of Medicine, No. 639, Zhizaoju Road, Huangpu District, Shanghai 200011, China

**Keywords:** Methylene blue, Nanoparticle, Dissolving microneedles, Lymphatic vessel, Imaging tracer

## Abstract

The lymphatic system serves many more functions than simply maintaining tissue fluid homeostasis, and its structural and functional changes indicate the occurrence of disease. Current clinical methods for the assessment of the lymphatic system, however, are severely limited because of their nontargeting ability, invasiveness, high cost, and radiation risk. Herein, we propose a simple and painless method for visualizing and quantifying the lymphatic system. This method is based on the noninvasive administration of a novel lymphatic tracer via dissolvable microneedles, followed by the application of a portable detection device for near-infrared (NIR) imaging. The tracer is prepared by incorporating the clinically approved NIR fluorescent dye methylene blue (ME) into the nanomaterial monomethoxyl poly(ethylene glycol)-*b*-poly(ε-caprolactone) (MPEG-PCL@ME). This novel tracer displays superior fluorescence properties, stability, biocompatibility, and targeting features in comparison with ME solution alone. Lymphography with MPEG-PCL@ME *in vivo* clearly revealed the lymphatic vessel morphology. Notably, compared with ME and indocyanine green, MPEG-PCL@ME can easily identify the dominant lymphatic vessels and nodes in rats with higher imaging quality. Furthermore, a series of segmental contracting sections are detected with MPEG-PCL@ME, allowing straightforward identification of the lymphatic pump, which provides direct evidence for exquisitely evaluating lymphatic functions.

## Highlights

- A novel lymphatic vessel imaging tracer, monomethoxyl poly(ethylene glycol)-*b*-poly(ε-caprolactone)@methylene blue (MPEG-PCL@ME), was developed by incorporating a clinically approved near-infrared (NIR) fluorescent dye, ME, into nanomaterials.
- Compared with ME and indocyanine green solutions, MPEG-PCL@ME displayed improved targeting ability, stability, optical quality, and biocompatibility.
- A straightforward and noninvasive method based on this tracer that can be administered intradermally via microneedles and can utilize imaging from a portable detection device to visualize and quantify the lymphatic system is proposed.

## Background

The lymphatic system is a complex network of lymphatic vessels and lymph nodes that serves essential roles in tissue fluid homeostasis and immunological surveillance in the human body [1–5]. Changes in lymphatic system function and structure are related to the occurrence of many diseases, such as lymphoedema, which results from fluid accumulation in tissues and can lead to the development of swelling, chronic inflammation, and even disability [6–8]. Additionally, other disorders, including venous insufficiencies, tumour development and metastasis, and impaired wound healing, are closely associated with alterations in the lymphatic system [9–11]. Thus, it is crucial to assess the lymphatic vessel structure and function for early diagnostic and treatment purposes in the clinic. Despite the fact that numerous techniques, such as computed tomography, magnetic resonance imaging, and positron emission tomography, have been utilized to assess lymphatic system function, their routine application in the clinic is severely limited because of their complexity, nontargeting nature, invasiveness, and radioactively labelled tracer risks [12–16]. Lymphatic-specific techniques [e.g. lymphoscintigraphy and indocyanine green (ICG) lymphography] have also been shown to be effective for evaluating the structure and function of the lymphatic system. Nevertheless, these methods present drawbacks in terms of dynamic functional evaluation, limited spatial resolution, and the potential for allergic reactions to certain contrast agents. Therefore, developing a method for safely and qualitatively identifying structural and functional alterations of the lymphatic system is highly desirable.

Near-infrared (NIR) imaging has recently emerged as an attractive minimally invasive method for visualizing the lymphatic system. Moreover, the replacement of radioactive tracers with fluorescent dyes for lymphatic system imaging and evaluation can eliminate the limitations of radioactivity and minimize costs [17–21]. Generally, an ideal fluorescent tracer for lymphatic system imaging should display the following characteristics: (1) rapid absorption by tissue and clear lymphatic vessel imaging capabilities; (2) targeting of lymphatic vessels without entering capillaries, to prevent massive tissue staining; and (3) low toxicity and low cost [22–25]. Methylene blue (ME), a clinical fluorescent dye approved by the European Medicines Agency (EMA) and the Federal Drug Administration (FDA), has been extensively adopted for parathyroid gland identification, pancreatic tumour imaging, and breast cancer sentinel node biopsy [26–29]. Because of its optical properties and favourable safety profile—which includes the absence of issues related to ICG iodine allergies or radionuclide radiation hazards—ME has the potential to serve as a lymphatic tracer for lymphatic system imaging and quantification analysis [30–32]. However, ME has certain limitations. It is unstable and tends to aggregate in aqueous solutions, resulting in decreased fluorescence. Furthermore, ME lacks specific lymphatic targeting properties because of its small particle size (<10 nm), which facilitates infiltration into capillary vessels, making lymphatic visualization challenging [33, 34]. This limitation may be addressed by incorporating ME into nanomaterials that exhibit a special degree of lymphatic tropism. On the other hand, nanomaterials possess a controlled size (10–100 nm), enabling improved lymphatic vessel targeting. Additionally, the encapsulation of ME within nanomaterials may increase therapeutic efficacy and reduce toxicity [35–37].

Another major challenge concerns the spatially controlled delivery of lymphatic tracers to specific skin layers. Since the dermal layer hosts a large number of lymphatic vessels, it would be most beneficial to deliver tracers directly to this layer to evaluate lymphatic vessel function [38, 39]. However, conventional intradermal injections, performed with hypodermic needles, require professionals to administer and cause severe pain to patients. Microneedles, which are composed of dozens of micron-sized needles, have garnered considerable attention as a potential delivery method since they can physically penetrate the outermost layer of the skin and deliver substances into the skin at a certain depth. Owing to their convenience of operation, painlessness, and good biocompatibility, MN-based injection tools represent an ideal option for delivering imaging tracers into the dermal interstitial space for lymphatic imaging [40–45]. Nevertheless, far less research has been conducted on microneedles in the field of biomedical imaging.

**Figure 1:**
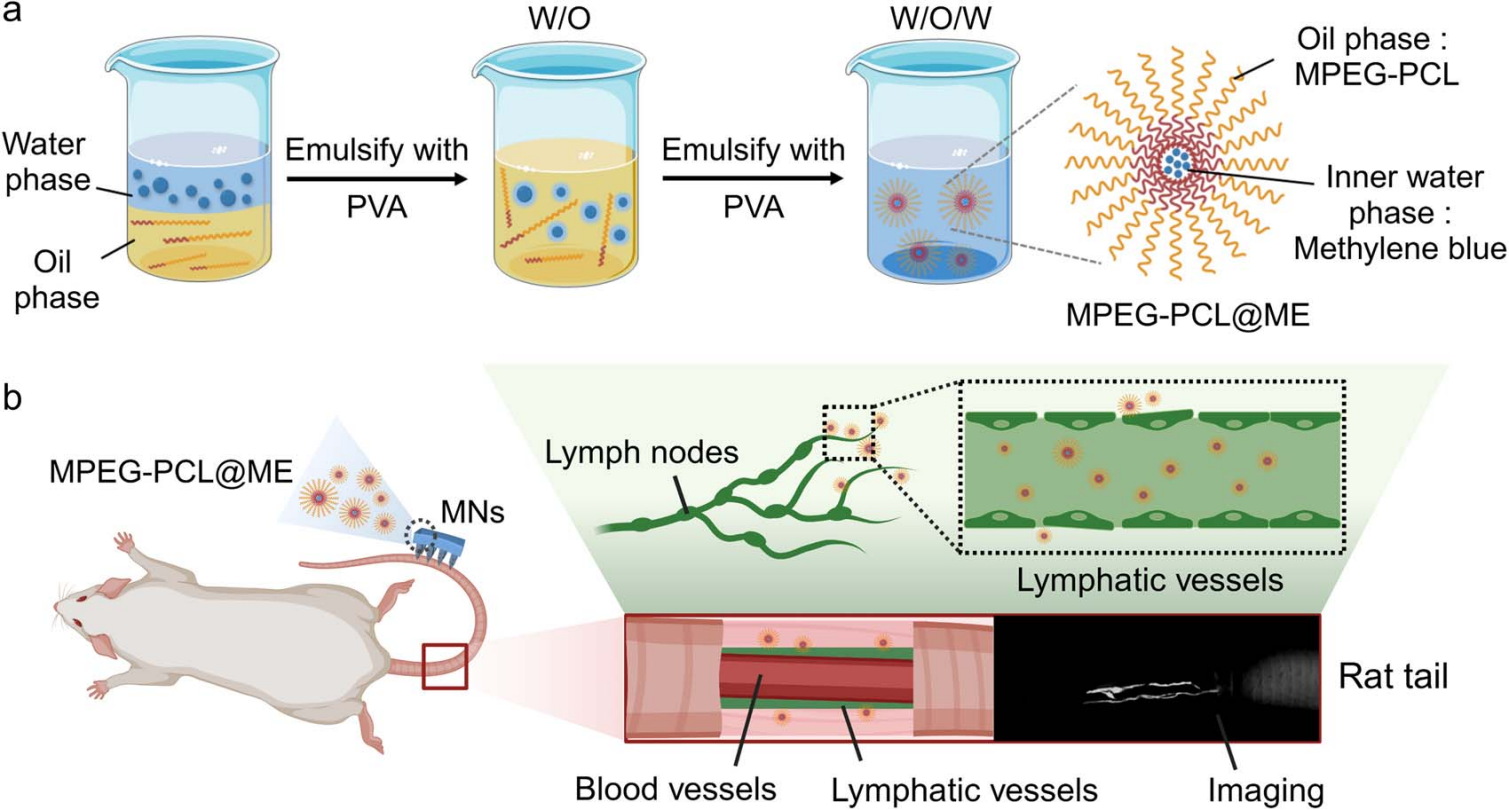
*In vivo* imaging of the lymphatic system using MPEG-PCL@ME-loaded MNs. (**a**) Schematic diagram of MPEG-PCL@ME synthesis. (**b**) *In vivo* imaging of normal lymphatic system and schematic route of lymphatic uptake of MPEG-PCL@ME. *PVA* polyvinyl alcohol, *MPEG-PCL* methoxy polyethylene glycol–polycaprolactone, *MPEG-PCL@ME* methoxy polyethylene glycol–polycaprolactone loaded methylene blue, *MNs* microneedles

In this work, a novel ME-based tracer [monomethoxyl poly(ethylene glycol)-*b*-poly(ε-caprolactone)@methylene blue (MPEG-PCL@ME)] that can be administered intradermally via microneedles for visualization and quantification of the lymphatic system in a noninvasive manner (Figure 1) was developed by a double emulsion method. Compared with a ME solution, MPEG-PCL@ME exhibits superior fluorescence properties, stability, biocompatibility, and targeting features. Structural and functional properties of the lymphatic system were assessed *in vivo* using microneedles, which revealed that the use of MPEG-PCL@ME as a tracer may provide a more exquisite representation of lymphatic vessel morphology and enable the identification of lymphatic vascular functions. Overall, this work proposes an efficient and noninvasive imaging approach for demonstrating the structures and functions of the lymphatic system with great accuracy and deep penetration, thus satisfying the growing demand for early disease diagnosis.

## Methods

### Materials

All the chemicals can be found in the online supplementary material unless otherwise noted and were used without additional purification.

### Preparation of MPEG-PCL@ME

Firstly, the MPEG-PCL copolymer was synthesized using ring-opening polymerization of ɛ-CL and MPEG5000. The procedures for the fabrication of MPEG-PCL copolymers are described in detail in the online supplementary material. Then, ME (1 mg) was dissolved in a 5% glucose solution (1 mL) to prepare stock solutions for further use. To prepare ME nanoparticles, stock solutions were diluted into different concentrations with the 5% glucose solution and then added dropwise into a dichloromethane solution (4 ml) containing MPEG-PCL (10 mg) for ultrasonic mixing. The mixture solution was then added into phosphate-buffered saline (PBS, 6 ml) containing 3% polyvinyl alcohol (PVA) dropwise under ultrasonication for 5 minutes. The ultrasonic mixed solution was poured into PBS (20 ml) containing 0.3% PVA and magnetically stirred for 12 hours. The excessive substances were removed by centrifugation (4000 rpm) and rinsed three times with water to produce the desired ME nanoparticles (abbreviated as MPEG-PCL@ME).

### Preparation of MPEG-PCL@ME microneedles

The dissolvable microneedles were fabricated from biodissolvable (water-soluble) materials (polymers), which can dissolve and be biodegraded in tissue fluid. Briefly, dissolvable microneedles loaded with MPEG-PCL@ME (abbreviated as MPEG-PCL@ME microneedles) were fabricated using an improved template method. The template of microneedles consisted of micron-sized needles that were arranged in a 15 × 15 array, with the height of each needle being 900 μm. To fabricate the MPEG-PCL@ME microneedles, 200 μl pre-solutions of MPEG-PCL@ME and hyaluronic acid (HA) (10 wt%) were first deposited into the template. The template was then vacuumed under negative pressure and placed overnight at room temperature in a desiccator. After complete desiccation, a reservoir was created via polydimethylsiloxane mould to get MPEG-PCL@ME microneedles. For the fabrication of blank dissolving microneedles, no MPEG-PCL@ME was added into the HA solution.

### Characterization of MPEG-PCL@ME

The morphology of MPEG-PCL@ME was characterized by a field emission scanning electron microscope (FM-SEM, FEI Quanta 250) and transmission electron microscope (TEM, TALOS F200X), respectively. The particle size and zeta potential of MPEG-PCL@ME were measured on nanoparticle size potentiometer (Zetasizer Nano ZS90). The infrared spectrum was obtained by using Fourier transform spectrometer (FTIR, Bruck EQUINOX55). The optical properties were recorded on UV–vis spectrophotometer (Evolution 201), luminescence spectrometer (LS 55, PerkinElmer), and IVIS spectrum imaging system (IVIS Spectrum, PerkinElmer).

### Characterization of MPEG-PCL@ME microneedles

The morphology of MPEG-PCL@ME microneedles were examined using an FM-SEM (FEI Quanta 250), a stereoscopic microscope (M125, Leica), and a fluorescence microscope (Olympus IX73), respectively.

### Mechanical property test of MPEG-PCL@ME microneedles

The mechanical property of MPEG-PCL@ME microneedles was evaluated with universal testing equipment (Zwick/Roell Z020). The MN sample was fixed onto an aluminium plate with needles facing upwards. A cylindrical probe made of flat-headed stainless steel was then used to apply a force on the microneedles in a vertical direction at a constant displacement speed of 0.01 mm s^−1^. Measurements were performed until a predefined displacement of 400 μm was reached.

### Release study of MPEG-PCL@ME microneedles *in vitro*

A vertical Franz diffusion cell system (Shanghai Kaikai Technology Trading Co., Ltd, China) was used to conduct the MPEG-PCL@ME release examination. The effective area of the vertical Franz diffusion cell system was 1.766 cm^2^. The harvested skin was placed between the donor and acceptor compartments and the microneedles were inserted into the skin. PBS was added to the acceptor compartment, which was then kept in a water bath at 37°C while being constantly stirred. After predefined time intervals, 1 ml of solution was removed from the acceptor chamber and replaced with an equivalent volume of PBS solution. The absorbance of the collected solutions was measured using an ultraviolet–visible (UV–Vis) spectrophotometer (Evolution 201). The cumulative amount of MPEG-PCL@ME release was determined by equation (1) in the online supplementary material.

### Cell cultures

Mouse lymphatic endothelial cells (LECs) were purchased from Shanghai Kui Sai Biotechnology Co., Ltd. LECs were grown in endothelial cell growth medium (PromoCell) supplemented with 20% foetal bovine serum (FBS) and 1% PS and cultured in an incubator (37°C, 5% CO_2_).

### Biocompatibility of MPEG-PCL@ME *in vitro*

The live/dead staining assay on LECs was conducted in 24-well plates with 1 × 10^5^ cells per well. After the different treatments, cells were stained with a mixed solution containing calcein acetoxymethyl ester/propidium iodide (calcein AM/PI, 2/8 μM) for 40 minutes, and photographs were taken using a fluorescent microscope (Olympus IX73).

### Hemolysis testing

The hemolysis ratio was tested by red blood cells (RBCs) from entire blood of C57BL/6 mice. Triton X-100 and normal saline were set as positive and negative controls, respectively. Various concentrations of MPEG-PCL@ME and the positive and negative groups (1 ml) were centrifuged for 15 minutes at 3000 rpm after being equilibrated in diluted RBC solutions (20 μL) for 2 hours at 37°C.

Various concentrations of MPEG-PCL@ME and the positive and negative groups (1 ml) were equilibrated in dilute RBC solutions (20 μL) at 37°C for 2 hours and centrifuged (3000 rpm) for 15 minutes. The percentage of hemolysis was estimated by measuring the absorbance of the supernatant solution at 545 nm using a UV–Vis spectrophotometer (Evolution 201). The hemolysis ratio was calculated using equation (2) in the online supplementary material.

### MPEG-PCL@ME uptake test

LECs were seeded at a density of 2 × 10^5^ cells per well onto transwell inserts (Corning) and cultured in endothelial cell growth medium overnight. Afterwards, LECs were incubated with MPEG-PCL@ME and uptake was assessed by fluorescence microscopy and flow cytometry. For the fluorescence microscopy experiment, cells were fixed with 4% paraformaldehyde (PFA) for 20 minutes and stained with F-actin reagent in accordance with the standard protocol. A fluorescent microscope (Olympus IX73) was used to capture the pictures. Cells were fixed with 2% PFA and then analyzed by flow cytometry (BD Biosciences).

### MPEG-PCL@ME transport experiment

MPEG-PCL@ME transport across LECs was assessed using a transendothelial transport model *in vitro*. Briefly, LECs were grown in endothelial cell growth medium for 48 hours on the bottom of transwell inserts. After that, the apical side of LECs was treated with MPEG-PCL@ME, and samples were taken from the basolateral compartment every 2 hours for up to 24 hours. The effective permeability was calculated by equation (3) in the online supplementary material.

### Animals

Sprague Dawley (SD) rats (male, 151–200 g) were acquired from SLAC Laboratory Animal Co., Ltd (Shanghai, China). All the animal experiments were conducted in accordance with the guidelines authorized by the Ethical Committee for Animal Experiments of Shanghai Jiao Tong University (China, A2023213).

### *In vivo* lymphatic vessel imaging

Rats were anaesthetized with isofluorane before and throughout operations, and their vital signs were continuously monitored. Then, a 1 cm circumferential incision was made for imaging. ME and ICG with a syringe and MPEG-PCL@ME microneedles were intradermally injected into the tail. The lymphatic vessel imaging was assessed by a portable imaging equipment.

### Biocompatibility test *in vivo*

Haematoxylin and eosin (H&E) staining was carried out to assess the toxicity of the material in conventional organs (lung, kidney, liver, spleen, and heart). A blood routine was performed to evaluate the biosafety of the material.

### Statistical analysis

All the data values are presented as the mean ± standard deviation (SD). GraphPad Prism Software (version 9) was used to analyse the between-group differences in experiments by a *t*-test (two-tailed), where the statistical significance was defined as ^*^P < 0.05, ^**^P < 0.01, ^***^P < 0.001, ^****^P < 0.0001, and ns (not significant *vs* the indicated group), respectively. For experiments with a single independent variable, one-way ANOVA was used to determine significant differences between groups, where the statistical significance was defined as ^*^P < 0.05, ^**^P < 0.01, ^***^P < 0.001, and ^****^P < 0.0001, respectively.

## Results

### Characterization of ME

**Figure 2:**
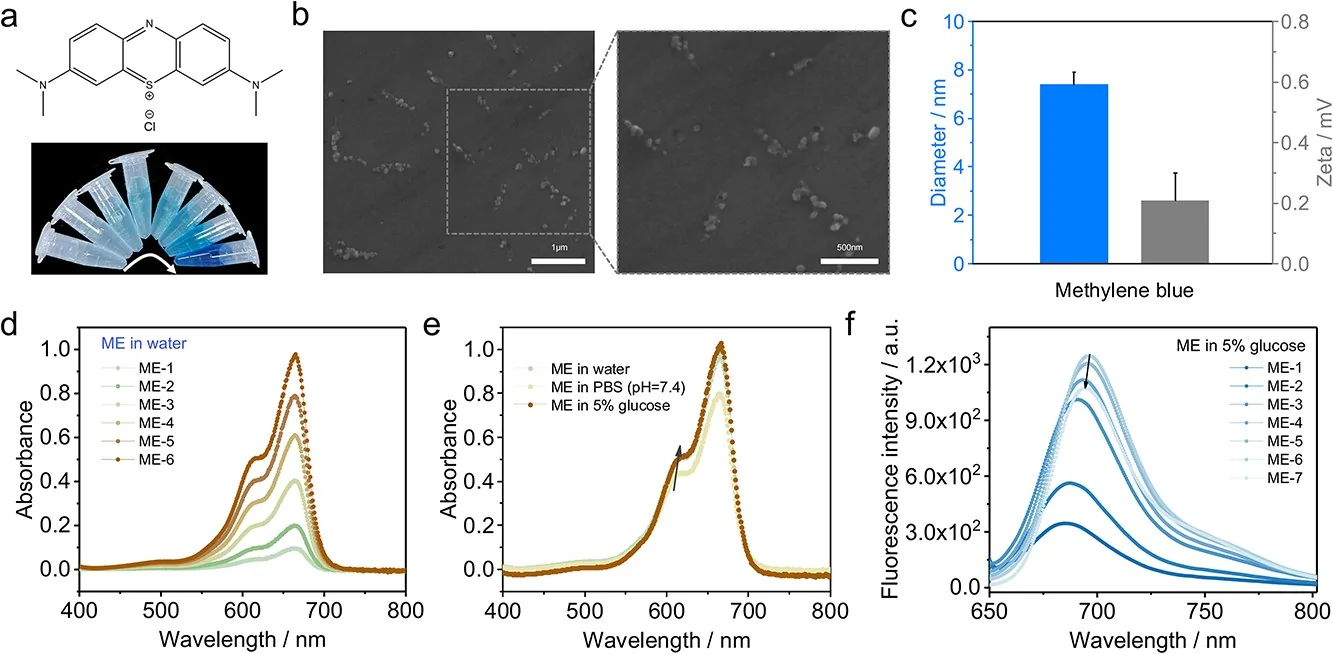
Characterization of ME. (**a**) Molecular structure of ME (top) and digital images of ME solutions at various concentrations (bottom) demonstrating a concentration-dependent visual gradient. (**b**) A representative SEM image and its locally enlarged image of ME. Scale bar: 1 μm, 500 nm. (**c**) DLS and zeta-potential data of ME. (**d**) Absorption spectra of ME at various concentrations in a water solution. (**e**) Absorption spectra of ME in water, PBS (pH = 7.4), and 5% glucose solution. (**f**) Fluorescence spectra of ME with different concentrations in 5% glucose solution. *ME* methylene blue, *DLS* dynamic light scattering

ME has been extensively used as a chromogenic dye for the diagnosis and treatment of several disorders, including cervical cancer, thyroid cancer, and breast cancer, and for the localization of other sentinel lymph nodes. Furthermore, when exposed to specific excitation light, ME may be employed as a fluorescent material to achieve real-time dynamic visualization of lymphatic structures without the radioactive risks of radionuclides or the iodine allergy issues associated with ICG. The chemical formula of ME, a urine-excretable fluorophore, and various concentrations of ME in water are presented in Figure 2a. The concentration-dependent visual variations of the ME solutions can be clearly observed. Field emission scanning electron microscopy analysis showed that ME exhibits an aggregated irregular morphology in aqueous solution (Figure 2b). The particle size and zeta potential of ME were characterized. As shown in Figure 2c, the average diameter of ME was ~7.39 nm, with a surface potential of 0.21 mV (Figure S1). However, the particle size and surface charge characteristics of ME greatly affect its entry into lymphatic vessels, since substances with small particles enter blood vessels more easily, whereas positively charged particles are more likely to be stored in the interstitium and are difficult to absorb for lymphatic vessels [46]. Afterwards, optical characterization of ME was performed. Both the absorption spectra and luminescence spectra of ME in water, PBS, and 5% glucose were obtained. As shown in Figure 2d, ME presented an absorption peak at 665 nm with a shoulder at 615 nm in water and 5% glucose (Figure S2). The absorption spectra of ME in PBS exhibited a similar envelope to that of water and 5% glucose, while the peak absorption wavelength of ME in PBS was slightly blueshifted, with an absorption peak at 663 nm and a shoulder peak at 610 nm (Figures 2e and S3). Moreover, the absorption peak increased with increasing concentrations of ME, but the absorption peak of ME in PBS was slightly lower than those in water and 5% glucose at the same concentration. The fluorescence peak of ME in various aqueous solutions ranged from 686 to 700 nm with increasing concentration (Figures 2f and S4). ME is unquestionably a typical NIR-I fluorescent dye given that the majority of its fluorescence falls within the NIR-I spectral range. Nevertheless, as the concentration of ME increased, the fluorescence emission peak of ME initially ascended and then declined. This can be attributed to the fact that ME exhibits a typical aggregation-induced quenching phenomenon at high concentrations, resulting in a decrease in fluorescence intensity. Moreover, methylene blue is prone to reduction into a non-luminous leuco-state due to its chemical instability in aqueous environments. Previous research indicated that a 5% glucose solution can effectively stabilize ME and enhance its optical properties compared to pure water; hence, glucose was selected as the solvent for subsequent research.

### Preparation and characterization of MPEG-PCL@ME

To overcome the above-mentioned limitations, ME was incorporated into nanoparticles by the double emulsion method for better lymphatic imaging. MPEG-PCL copolymers frequently possess a negative charge, which helps decrease nonspecific interactions with negatively charged cells and plasma proteins in the bloodstream. Additionally, MPEG-PCL self-assembly produces nanoparticles with modest sizes (<100 nm), facilitating initial entry into the lymphatics via the fenestrated endothelium and allowing better lymphatic targeting.

**Figure 3:**
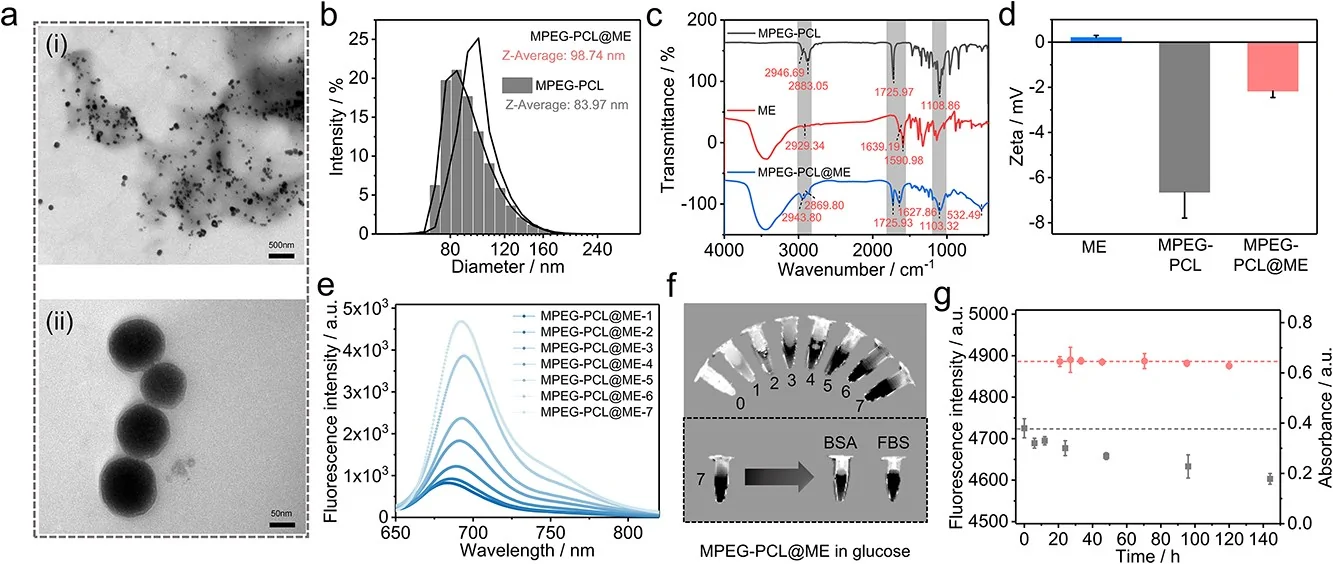
Characterization of MPEG-PCL@ME. (**a**) A representative TEM image (i) and its locally enlarged image (ii) of MPEG-PCL@ME. Scale bar: 500 nm. (**b**) Size distribution of MPEG-PCL and MPEG-PCL@ME. (**c**) FTIR spectrum of ME, MPEG-PCL, and MPEG-PCL@ME. (**d**) DLS data of ME, MPEG-PCL, and MPEG-PCL@ME. (**e**) Fluorescence spectra of MPEG-PCL@ME with different concentrations. (**f**) Fluorescence images of MPEG-PCL@ME at various concentrations. (**g**) Stability of MPEG-PCL@ME as assessed by absorbance and fluorescence monitoring. *ME* methylene blue, *PVA* polyvinyl alcohol, *MPEG-PCL* methoxy polyethylene glycol–polycaprolactone, *MPEG-PCL@ME* methoxy polyethylene glycol–polycaprolactone loaded methylene blue, *DLS* dynamic light scattering

A schematic diagram of the preparation process is shown in Figure 1a [47, 48]. The structure and copolymer distribution of the MPEG-PCL copolymer were characterized by Fourier transform infrared (FTIR) spectroscopy, nuclear magnetic resonance (^1^H-NMR) spectroscopy, and gel permeation chromatography (GPC). The FTIR results revealed that the characteristic absorption peaks at 2800–3000 and 1108.86 cm^−1^ correspond to the stretching vibrations of methylene groups (–CH) and C–O–C, respectively, of the MPEG block. In addition, other absorption peaks at 1725.97 and 3467.38 cm^−1^ were recorded from the MPEG-PCL copolymer, which relate to C=O and –OH stretching vibrations in the PCL block, respectively (Figure S5). The ^1^H-NMR spectrum of MPEG-PCL is shown in Figure S6. The characteristic methylene proton peak (−CH_2_) of the MPEG block is assigned to a peak at 3.67 ppm. The chemical shift at 4.07 ppm corresponds to CH_2_ of the caprolactone adjacent to the C=O in the PCL block. Other hydrogen resonances of the CH_2_ residues of the PCL block were detected at 2.31, 1.65, and 1.37 ppm. A relatively weak peak was found at 4.23 ppm, which is related to −OCH_2_CH_2_O− at the MPEG and PCL junctions. This indicates the successful synthesis of the MPEG-PCL copolymer and is consistent with the FTIR results. The GPC chromatogram of the MPEG-PCL copolymer revealed a symmetric unimodal peak with low polydispersity, which further confirmed that the as-prepared copolymer was formed via chemical bonding between the MPEG block and the PCL block rather than being a simple mixture of these two compounds (Figure S7). ME was subsequently loaded into the MPEG-PCL copolymer (MPEG-PCL@ME). The synthesized MPEG-PCL@ME had a near-spherical shape with good particle size monodispersity and a size of ~90 nm according to transmission electron microscopy (TEM) images (Figure 3a). Based on the results of dynamic light scattering, the particle size of MPEG-PCL@ME increased, with an average diameter of ~98.74 nm in comparison with MPEG-PCL, which is consistent with TEM findings (Figure 3b). Other common polymers, such as PLGA–PLA, PLGA–PVA, and PLA–PEG, were also chosen for comparison. ME was encapsulated in PLGA–PLA, PLGA–PVA, and PLA–PEG, with average diameters of 180.75, 204.68, and 160.53 nm, respectively, which are greater than those of MPEG-PCL@ME (>100 nm), indicating difficulty for these materials to enter lymphatic vessels (Figure S8). The IR spectra revealed strong absorption peaks at 2943.80, 1729.93, and 1103.32 cm^−1^, which are attributed to methylene–amide (−CH/−CH_2_), C=O, and C–O–C stretching vibrations, respectively, of the MPEG-PCL block, whereas after ME loading, the typical absorption peaks of ME were observed, including peaks at 1627.86 and 532.49 cm^−1^ assigned to the skeleton vibration peaks of the benzene ring and C–S–C, confirming that MPEG-PCL@ME was prepared successfully (Figure 3c). Moreover, in transitioning from ME to MPEG-PCL@ME, the zeta potential changed from 0.21 ± 0.09 to −2.16 ± 0.28 mV, which was suitable for further study, as the zeta potential shifted negatively and further proved that the ME was successfully loaded into MPEG-PCL (Figure 3d).

The optical properties of MPEG-PCL@ME were subsequently investigated. Both the MPEG-PCL and the ME characterization peaks could be perceived in the MPEG-PCL@ME absorption spectrum, and the UV–Vis absorption envelope was consistent across the various MPEG-PCL@ME concentrations (Figure S9). Moreover, a slight blueshift in the MPEG-PCL@ME absorption peak compared with that of the free ME aqueous solution indicated an interaction between MPEG-PCL and ME. The fluorescence intensity of MPEG-PCL@ME was greater than that of ME, and it increased with increasing concentration, as revealed by the fluorescence spectra. Even at higher concentrations, there was no abrupt decrease in fluorescence intensity (Figures 3e and S10). This finding was also supported by the fluorescence signal monitored through the IVIS spectrum system: the fluorescence intensity was positively correlated with increasing concentration (Figure 3f). Moreover, there was no discernible decrease in the fluorescence signal following MPEG-PCL@ME incubation with FBS and bovine serum albumin (BSA), which were absent from the free ME (Figure S11). According to these observations, the complexing of MPEG-PCL and ME limited the intramolecular interaction (i.e. aggregation) of ME in aqueous solution. This prevented oxidation and inhibited fluorescence quenching, hence increasing the intensity of fluorescence. Although ME is unstable in aqueous solution, its chemical stability was greatly improved when prepared as MPEG-PCL@ME (Figure 3g).

### Biocompatibility and transport of MPEG-PCL@ME *in vitro*

**Figure 4:**
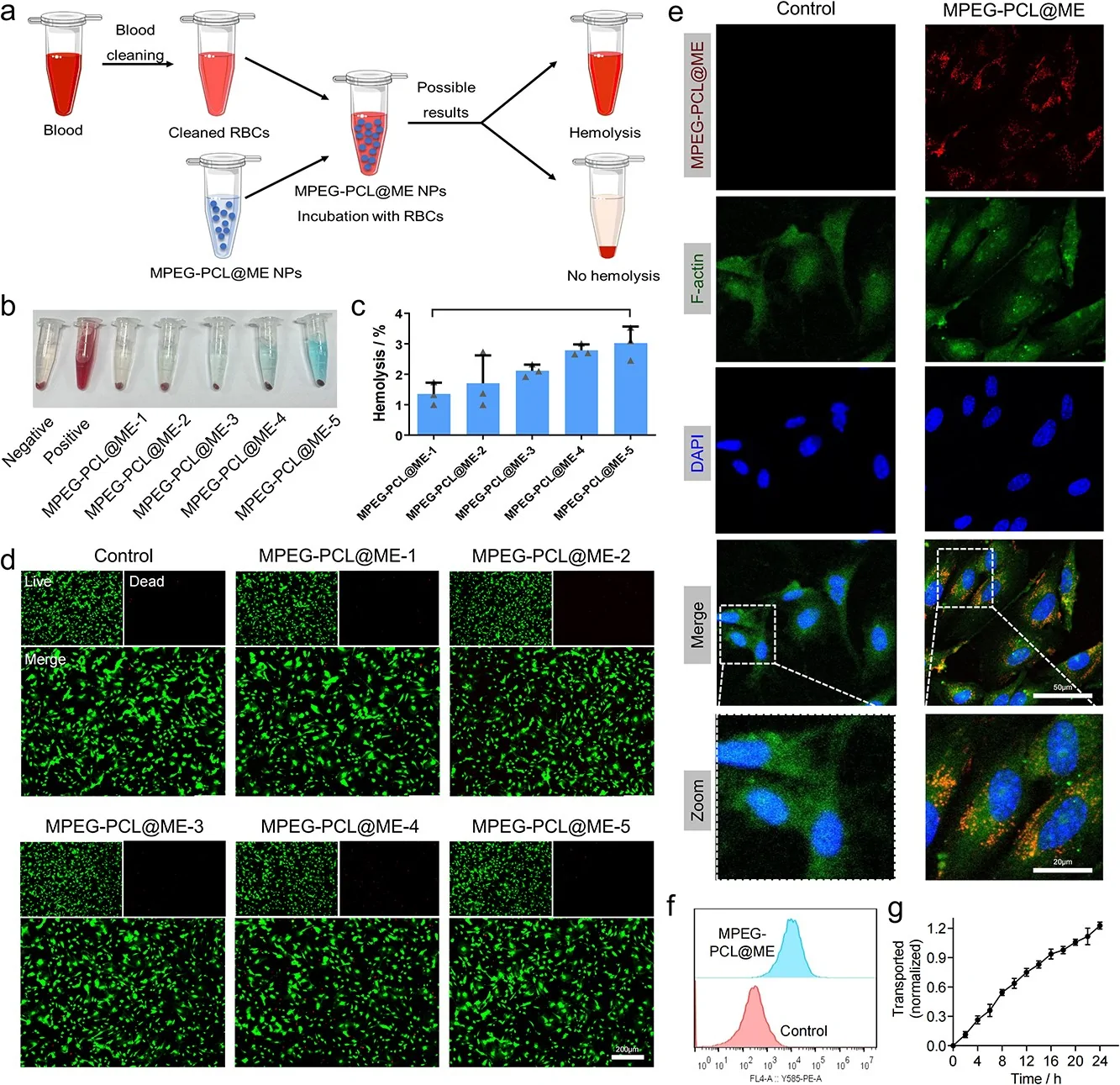
Biocompatibility and uptake of MPEG-PCL@ME. (**a**) Schematic diagram of the hemolysis experiment. (**b**) Photographs of the hemolysis experiment of MPEG-PCL@ME with various concentrations. (**c**) The hemolysis rate of MPEG-PCL@ME at various concentrations. (**d**) Fluorescence images of the live/dead experiment of MPEG-PCL@ME at various concentrations. Scale bar: 200 μm. (**e**) Confocal fluorescence image of LECs stained for F-actin (green) and DAPI (blue) treated with MPEG-PCL@ME (red). Scale bar: 50 μm and 20 μm in the zoomed images. (**f**) Flow cytometry results of MPEG-PCL@ME within LECs. (**g**) Percentage of MPEG-PCL@ME transported across LEC over time. All data are presented as the mean ± SD. Differences among the groups were examined with one-way ANOVA. ^*^P <0.05, ^**^P < 0.01, ^***^P < 0.001, ^****^P < 0.0001, and ns (not significant versus the indicated group). *ME* methylene blue, *MPEG-PCL@ME* methoxy polyethylene glycol–polycaprolactone loaded methylene blue

A key component of biomaterials for prolonged application is their exceptionally high degree of biocompatibility. To examine the biosafety of MPEG-PCL@ME following intravenous injection, a hemolysis experiment was conducted to assess the impact of MPEG-PCL@ME on RBCs. The entire experimental process is shown in Figure 4a, and Triton X-100 and normal saline were used as positive and negative controls, respectively. As shown in Figure 4b, images of RBCs incubated with various solutions for 2 hours at 37°C following centrifugation revealed no noticeable hemolysis effect, with the exception of the Triton X-100 group. Moreover, the hemolysis ratio of MPEG-PCL@ME at various dosages was <5%, which contributed to the extended circulation duration for intravenous injection (Figure 4c). The results of the low hemolysis ratio showed that MPEG-PCL@ME exhibited significant biosafety for intravenous injection *in vivo* and could be employed for imaging in further biological studies. In addition, a live/dead experiment was performed on mouse LECs to further verify the biocompatibility of MPEG-PCL@ME at the cellular level. In the representative images of live/dead staining, a high intensity of green fluorescence was displayed along with almost no or little red fluorescence (Figures 4d and S12). This observation is consistent with the previous results, indicating that all the MPEG-PCL@ME groups exhibited satisfactory biocompatibility. As a result, MPEG-PCL@ME-7 was chosen for follow-up study.

Next, using an *in vitro* transendothelial transport model in which a monolayer of LECs was cultivated on the bottom of a Transwell to simulate transport from the interstitium into the lymphatic vessel, the ability of MPEG-PCL@ME to be transported by lymphatics was evaluated. Fluorescence microscopy revealed that MPEG-PCL@ME was initially internalized by LECs, as evidenced by the intensity of its fluorescence, without compromising endothelial integrity (Figure 4e). Notably, MPEG-PCL@ME significantly colocalized with F-actin in LECs, suggesting its uptake by lymphatics (Figure S13). These results were further corroborated by flow cytometry, which revealed a substantial fluorescent signal attributable to MPEG-PCL@ME within LECs (Figure 4f). Subsequently, the transport ability of MPEG-PCL@ME across LECs was assessed. The results indicated that MPEG-PCL@ME was effectively transported across LECs over time, with an ∼10.8-fold increase in transport observed by 24 hours, increasing from 0.113 at 2 hours to 1.227 at 24 hours (Figure 4g). Consistent with these quantitative results, images collected at various time points also revealed a notable decrease in the intracellular fluorescence intensity of MPEG-PCL@ME (Figure S14). Collectively, these results indicate that MPEG-PCL@ME can be taken up and transported across lymphatics, highlighting its potential viability as a contrast agent for lymphatic vessel imaging.

### Preparation and characterization of MPEG-PCL@ME microneedles

**Figure 5:**
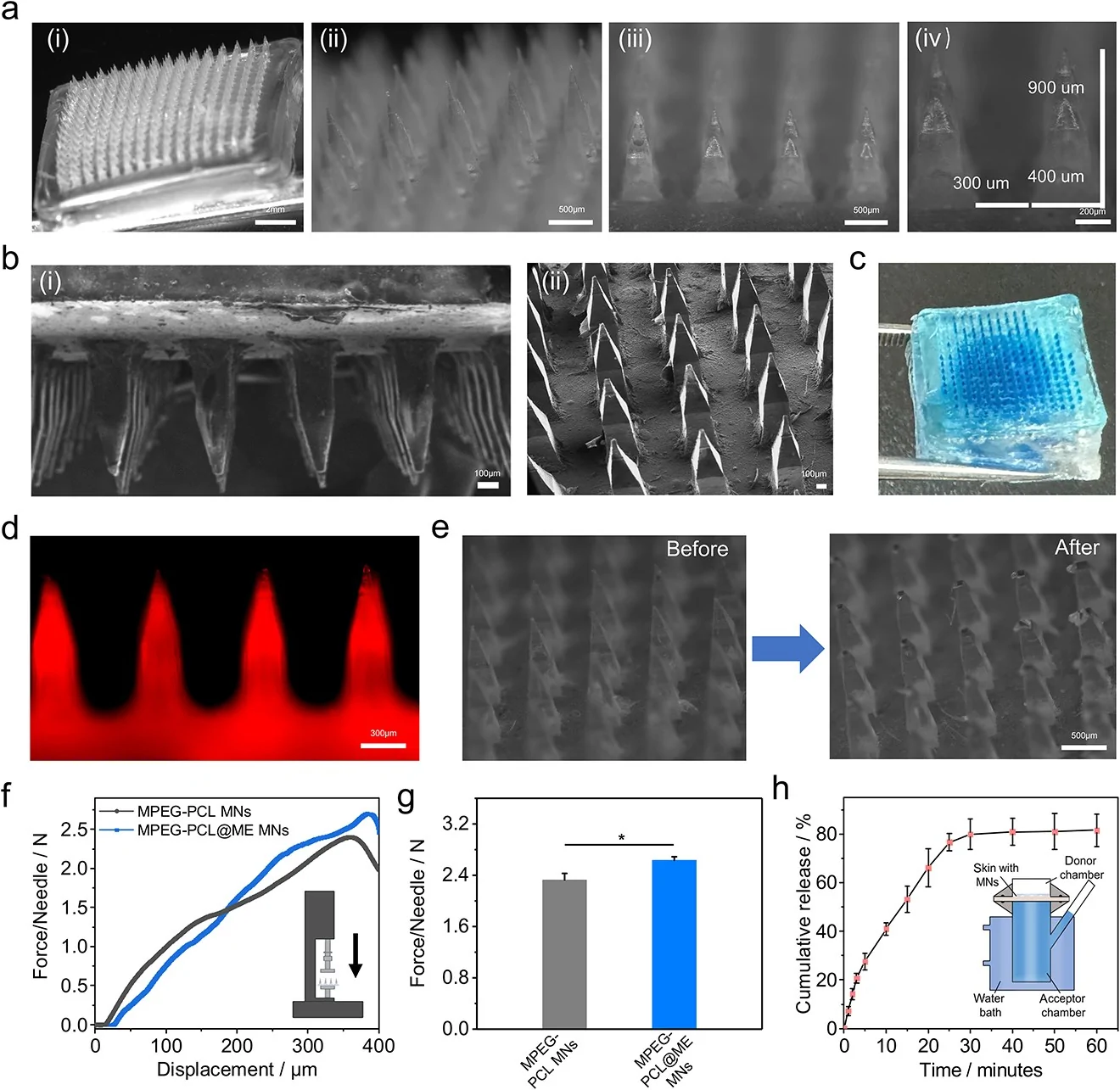
Preparation and properties of MPEG-PCL@ME MNs. (**a**) Representative photographs showing the complete (i) and its enlarged top (ii) and side (iii, iv) view of MPEG-PCL@ME MNs. Scale bar: 2 mm in (i), 500 μm in (ii, iii), and 200 μm in (iv). (**b**) SEM images of MPEG-PCL@ME MNs. Scale bar: 100 μm in (i, ii). (**c**) Photograph of MPEG-PCL@ME MNs loaded with MPEG-PCL@ME solution at the backing layer. (**d**) Representative fluorescence images of MPEG-PCL@ME MNs. Scale bar: 300 μm. (**e**) Two representative images showing the morphology of the MPEG-PCL@ME MNs before (left) and after (right) compression. Scale bar: 500 μm. (**f**) Compression curve and (**g**) mechanical strength of MPEG-PCL MNs and MPEG-PCL@ME MNs. (**h**) Cumulative release curve of MPEG-PCL@ME MNs and schematic diagram of the vertical Franz diffusion cell system (inserted picture). Differences among the groups were examined with a *t*-test (two-tailed). ^*^P < 0.05, ^**^P < 0.01, ^***^P < 0.001, ^****^P < 0.0001, and ns (not significant versus the indicated group). *ME* methylene blue, *MNs* microneedles, *MPEG-PCL MNs* methoxy polyethylene glycol–polycaprolactone microneedles, *MPEG-PCL@ME MNs* methoxy polyethylene glycol–polycaprolactone loaded methylene blue microneedles

Dissolving microneedles loaded with MPEG-PCL@ME (abbreviated as MPEG-PCL@ME microneedles) were fabricated by a template method. According to representative stereomicrographs, the overall appearance of the MPEG-PCL@ME microneedles was square-shaped, with each needle remaining intact and arrayed in a 15 × 15 array with a needle height of ~900 μm, tip-to-tip distance of 300 μm, and base diameter of 400 μm (Figures 5a and S15). Similarly, the morphological characteristics of the MPEG-PCL@ME microneedles were the same under scanning electron microscopy, and their needles were conical and quadrangular in shape (Figures 5b and S16). To further improve visualization, rhodamine was added during the MN creation procedure, and fluorescence micrographs revealed that the MPEG-PCL@ME microneedles were successfully separated from the mould and retained their entire tip morphologies (Figures 5d and S17).

Microneedles have become a popular noninvasive strategy for transdermal substance administration because of their unique benefits (painlessness, self-dosing, etc.). However, microneedles confer an extremely limited ability to transport substances because of their small size. In this case, a repository was set in place behind the microneedles, which could improve the effectiveness of substance transportation and ensure protection against substance leakage (Figure 5c). microneedles need to be sufficiently mechanically strong to be able to pierce the skin and deliver substances. Previous research indicated that a microneedle with a hardness of 0.1–0.2 N per needle could penetrate the stratum corneum layer. Therefore, the mechanical properties of MPEG-PCL@ME microneedles were tested by compression tests *in vitro*. Stereomicrographs of MPEG-PCL@ME microneedles following compression experiments revealed that the microneedles had been deformed by bending and compression, with no visible fragmentation (Figure 5e). According to the test findings, the force of each needle gradually increased as the displacement increased. However, the force abruptly decreased as the displacement approached 400 μm (Figure 5f). The mechanical forces of a single needle of blank microneedles without MPEG-PCL@ME (abbreviated as HA microneedles) and MPEG-PCL@ME microneedles were 1.98 and 2.52 N, respectively, when the displacement was 400 μm (Figure 5g). Both of these values are substantially greater than the minimum force required to pierce the stratum corneum layer, suggesting the feasibility of applying this type of microneedles in subsequent use. To further investigate the efficiency of microneedles in MPEG-PCL@ME delivery, *in vitro* penetration studies were performed on isolated skin using a vertical Franz diffusion cell system. The cumulative curve revealed that the release of MPEG-PCL@ME gradually increased and finally plateaued, with 80% cumulative release (Figure 5h). These results imply that these kinds ofmicroneedles could be used as convenient tools for the efficient delivery of MPEG-PCL@ME.

### Targeting lymphatic vessel imaging of MPEG-PCL@ME microneedles *in vivo*

**Figure 6:**
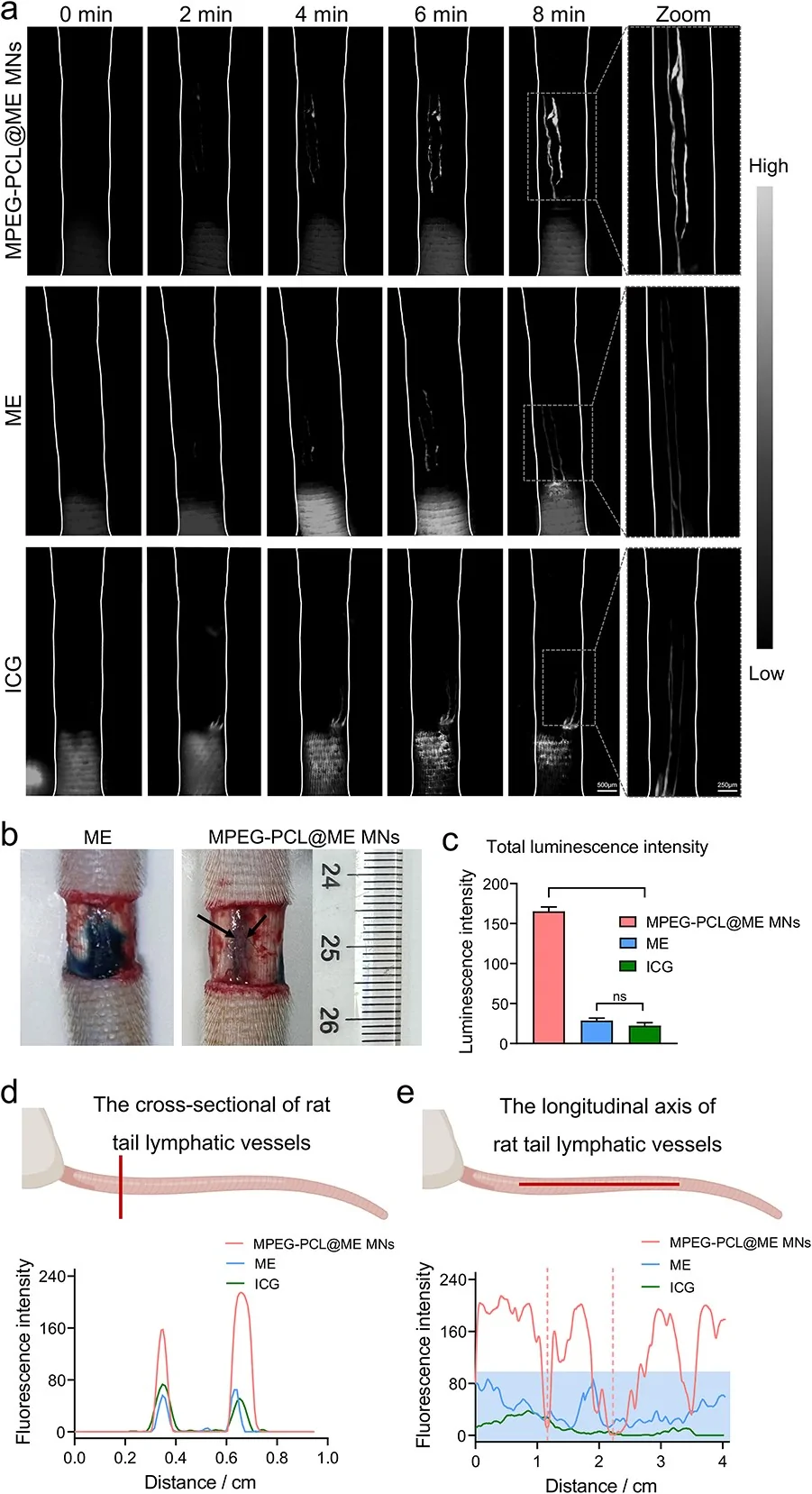
*In vivo* fluorescence images of MPEG-PCL@ME MNs in a rat tail targeting lymphatic vessels. (**a**) Fluorescence imaging of normal lymphatic vessels on one side of the rat tail after treatment with MPEG-PCL@ME MNs and injection of ME and ICG, respectively. Scale bar: 500 and 250 μm in the zoomed image (*n* = 3). (**b**) Photos of the rat tail after treatment with MPEG-PCL@ME MNs, ME, and ICG. (**c**) Quantitative analysis of fluorescence intensity in (a) (*n* = 3). Quantitative analysis of fluorescence intensity of (**d**) the cross-sectional level and (**e**) the longitudinal axis of rat tail lymphatic vessels of MPEG-PCL@ME MNs, ME, and ICG (*n* = 3). Differences among the groups were examined with one-way ANOVA and a *t*-test (two-tailed). ^*^P < 0.05, ^**^P < 0.01, ^***^P < 0.001, ^****^P < 0.0001, and ns (not significant versus the indicated group). *ME* methylene blue, *MNs* microneedles, *ICG* indocyanine green, *MPEG-PCL@ME MNs* methoxy polyethylene glycol–polycaprolactone loaded methylene blue microneedles

The *in vivo* imaging ability of the MPEG-PCL@ME microneedles was studied. First, the distribution of lymphatic vessels is described. There are four major lymphatic vessels in the tail of rats, which are arranged in pairs on both sides with an almost symmetrical distribution and are located lateral to the tail artery and vein. Since lymphatic vessels are colourless and transparent, they are difficult to visually identify by the naked eye. In addition, only two of the four lymphatic vessels could be shown in the images, as the lateral view was employed in the follow-up *in vivo* imaging experiments. Luminescence imaging was subsequently performed *in vivo* by intradermally injecting equal concentrations of ME and MPEG-PCL@ME microneedles into the rat tail. As shown in Figure 6a, ME swiftly entered and filled the lymphatic vessels along the rat tail, flowing from the injection site towards the proximal part of the rat tail 4 minutes after injection. However, ME imaging did not reveal clear morphology of the lymphatic vessels, and there was a significant amount of ME leakage at the skin margin within the excision site, which made it difficult to distinguish the lymphatic vessels (Figure 6b). Conversely, two lymphatic vessels were clearly visible following the application of MPEG-PCL@ME microneedles; nevertheless, the imaging time was longer than that of ME because MPEG-PCL@ME needed to be initially released from the microneedles. Additionally, two lymphatic vessels were visible in the excised skin photos, with virtually no MPEG-PCL@ME leakage, confirming the successful targeting of lymphatic vessels achieved by MPEG-PCL@ME microneedles. On the other hand, rats that received injections of the same quantity of ICG to the same location of the tail served as controls. As shown in Figure 6a, the outline of the two lymphatic vessels in the ICG group is not as clear as that in the MPEG-PCL@ME group. According to the statistical data, the luminescence intensity of MPEG-PCL@ME was at least three times greater than that of ME and ICG (Figure 6c). Thus, MPEG-PCL@ME microneedles can be employed as a lymphatic tracer for lymphatic vessel imaging, allowing for easier identification of lymphatic vessel morphology with higher imaging quality. In comparison with ME and ICG, microneedles can deliver contrast agents in a more convenient and painless manner.

The cross-sectional and longitudinal-axis luminescence intensities of the rat tail were subsequently analysed to provide information on the lymphatic vessels present. The cross-section of the rat tail in the MPEG-PCL@ME MN group displayed two signal peaks that corresponded to the locations of the two lymphatic vessels (Figure 6d). Although two peaks were likewise observed in the ME and IGC groups, their signals were significantly weaker than those of the MPEG-PCL@ME microneedles. Notably, the two peaks in the MPEG-PCL@ME MN group also differed in signal intensity: that is, one peak displayed a stronger signal, and the other was relatively weak. Previous reports have demonstrated that dominant lymphatic vessels possess higher signal intensity and transportation velocity because of the presence of preferential lymphatic drainage patterns [49, 50]. As a result, the vessel with higher signal intensity, as the predominant lymphatic vessel on lymphography, presented considerably greater transportation velocity. In contrast, no such significant difference was detected in the ME group. Additionally, the fluorescence signal intensity along the longitudinal axis of the rat tail lymphatic vessels in the MPEG-PCL@ME MN group presented a segmental pattern distribution (Figure 6e). Segmental contractions of lymphatic vessels, commonly referred to as ‘lymphatic pumps’, are responsible for lymph fluid drainage. Therefore, identifying the lymphatic pump in a series of segmental contracting sections in the MPEG-PCL@ME MN group by analysing the distribution of signal intensity throughout the rat tail lymphatic vessels was straightforward. Conversely, there was no discernible pattern in the distribution of segmental contracting sections along the lymphatic vessels in the ME and ICG groups.

To further validate the viability of MPEG-PCL@ME microneedles for lymphography, *in vivo* imaging of the lymphatic vessels of rat hind limbs was conducted since they morphologically vary from the tail. The morphology of the lymphatic vessels in the MPEG-PCL@ME, ME, and ICG groups is displayed in Figure S18A. Nevertheless, the images of the MPEG-PCL@ME MN group showed more precise structures, with significantly greater overall luminescence intensity and more prominent peaks of lymphatic vessels (Figure S18B). Notably, the segmental contracting section caused by the lymphatic pump was visible again in the MPEG-PCL@ME MN group (Figure S18C). Concurrently, the lymph node could be identified on the images of the MPEG-PCL@ME MN group. In contrast, the distribution of segmental contracting sections along the lymphatic vessel in the ME and ICG groups was random, with no discernible pattern. Consequently, compared with ME alone, MPEG-PCL@ME was superior to ME and ICG at the same concentration, indicating that MPEG-PCL@ME could more exquisitely represent lymphatic vessel morphology and identify lymphatic vascular functions more accurately, meeting the increasing demand for evaluating lymphangiographic function.

### Biosafety of MPEG-PCL@ME for lymphography

**Figure 7:**
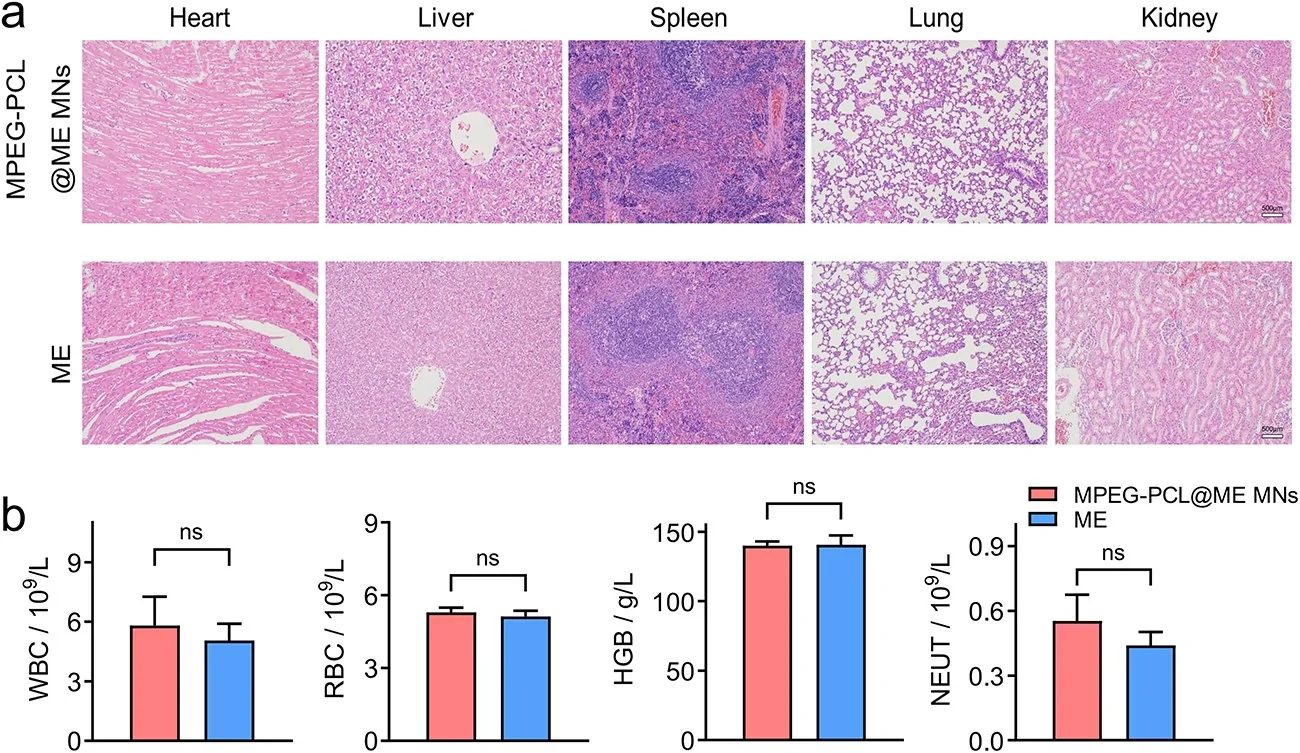
*In vivo* biosafety of MPEG-PCL@ME. (**a**) H&E staining of major organs (lung, liver, spleen, kidney, and heart) of the mice after different treatments. Scale bar: 500 μm. (**b**) Routine blood test following different treatments. Differences among the groups were examined with a *t*-test (two-tailed). ^*^P < 0.05, ^**^P < 0.01, ^***^P < 0.001, ^****^P < 0.0001, and ns (not significant versus the indicated group). *ME* methylene blue, *MNs* microneedles, *MPEG-PCL@ME MNs* methoxy polyethylene glycol–polycaprolactone loaded methylene blue microneedles, *WBCs* white blood cells, *RBCs* red blood cells, *HGB* haemoglobin, *NEUT* blood platelets, *LYMPH* lymphocytes

The biosafety of MPEG-PCL@ME during administration was evaluated *in vivo*. As shown in Figure 7a, H&E staining revealed no inflammatory infiltration or pathological changes in essential organs (lung, kidney, liver, spleen, or heart) following the injection of MPEG-PCL@ME microneedles. Moreover, all the blood parameters, including haemoglobin (HGB), neutrophils (NEUT), RBCs, and white blood cells (WBCs), were within the normal range according to the results of routine blood tests (Figure 7b). In conclusion, MPEG-PCL@ME indicated satisfactory biosafety and minimal cytotoxicity, suggesting that it could be used as a lymphatic tracer for long-term lymphography.

## Discussion

The present study demonstrated that encapsulating ME in MPEG-PCL nanoparticles and administering the formulation via dissolving microneedles yielded a NIR-I lymphatic tracer with improved physicochemical stability, enhanced lymphatic targeting, and favorable biosafety.

At the molecular level, free ME exhibits attractive NIR‑I fluorescence but suffers from aggregation‑induced quenching at high concentrations and chemical instability in aqueous environments, leading to decreased signal intensity and duration in vivo [51, 52]. Encapsulation within MPEG‑PCL nanoparticles markedly increased the fluorescence intensity of ME, prevented abrupt quenching even at higher concentrations, and maintained fluorescence stability in the presence of serum proteins such as FBS and BSA. These findings suggest that the polymeric matrix effectively restricts unfavourable intra‑ and intermolecular interactions of ME and protects it from conversion to its nonfluorescent leuco form, which is particularly important for continuous monitoring of lymphatic dynamics.

From the carrier design perspective, compared with alternative polymers such as PLGA, PLLA, chitosan, and liposomes, MPEG-PCL demonstrates a unique combination of advantages in terms of biocompatibility, biodegradability, and functionalization convenience, justifying its selection for drug delivery and biomedical applications. Its superior biocompatibility arises from the synergistic effects of its components: the hydrophilic MPEG segment forms a dense hydration layer that minimizes protein adsorption and opsonization, significantly extending blood circulation time through reduced RES recognition, whereas the PCL core, an FDA-approved polymer, exhibits low intrinsic toxicity [53, 54]. In contrast, PLGA and PLLA lack this efficient stealth ability, and their acidic degradation products (lactic/glycolic acid) can provoke inflammatory responses [55–57]. Liposomes, although biocompatible depending on their lipid composition, suffer from rapid RES clearance unless specifically PEGylated and may trigger complement activation [58]. Chitosan, while generally biocompatible, poses risks of mild allergenic reactions or dose-dependent cytotoxicity because of its cationic nature [59, 60]. In terms of biodegradability, MPEG-PCL offers controlled, slow degradation kinetics via ester hydrolysis of the PCL segment, yielding neutral, metabolizable 6-hydroxyhexanoic acid without creating an inflammatory acidic microenvironment [61, 62]. This makes it ideal for sustained long-term-release applications. Therefore, MPEG-PCL is selected on the basis of its material properties that govern its behaviour within the lymphatic system: PEG-mediated stealth reduces immune clearance; neutral, biocompatible degradation minimizes inflammatory responses; and a tuneable degradation rate enables reliable, sustained drug release. These advantages confer superiority over PLGA for lymphatic delivery, even when engineered to comparable sizes, as MPEG-PCL directly addresses the critical lymphatic challenges of longevity, biocompatibility, and controlled release. While its small size facilitates lymphatic entry, the intrinsic properties of MPEG-PCL ensure effective and sustained function within this microenvironment.

Regarding the delivery route, dissolving MNs helped overcome several drawbacks of conventional intradermal or subcutaneous injections in terms of patient comfort and compliance [63]. MPEG‑PCL@ME MNs demonstrated sufficient mechanical strength to reliably pierce the stratum corneum with forces far exceeding the minimal requirement, while maintaining structural integrity under compression. The inclusion of a backing-layer reservoir increased the drug‑loading capacity and transdermal delivery efficiency, enabling localized release of MPEG‑PCL@ME into superficial tissues at high concentrations and thereby promoting rapid entry into superficial lymphatic vessels while limiting non‑target exposure to the vascular system. The *in vitro* Franz diffusion data, showing approximately 80% cumulative release, further support the efficient delivery capability of this system.

Functionally, MPEG‑PCL@ME MNs provided superior lymphatic imaging performance compared with free ME and ICG in both tail and hind limb models. The MPEG‑PCL@ME group not only produced clearer delineation of lymphatic vessel morphology with substantially higher signal intensity but also revealed segmental fluorescence patterns along the longitudinal axis of the vessels that were consistent with lymphatic pump‑mediated segmental contractions. In addition, lymph nodes could be readily identified on the images. Conversely, our findings were consistent with previous reports: the free ME and ICG groups exhibited not only weaker fluorescence signals but also pronounced peripheral skin leakage, making it difficult to delineate lymphatic vessel morphology and function with high precision [64, 65]. These data suggest that MPEG‑PCL@ME MNs can achieve both high‑resolution structural imaging and functional assessment of lymphatic contractility and drainage pathways, which may be beneficial for evaluating lymphographic function, identifying dominant lymphatic routes, and monitoring disease‑related lymphatic dysfunction.

In terms of safety, MPEG‑PCL@ME exhibited low hemolysis, negligible cytotoxicity toward LECs, and no detectable histopathological abnormalities or haematological perturbations in major organs, supporting its potential for repeated or potentially long‑term applications in lymphography. Nonetheless, several limitations remain. The present work was conducted in healthy rat models; thus, the diagnostic and monitoring value of MPEG‑PCL@ME MNs in pathological conditions such as lymphedema or tumour‑induced lymphatic remodeling remains to be explored. Additionally, further optimization of dosing regimens, imaging time windows, and long‑term biodistribution and clearance is warranted to facilitate clinical translation.Overall, by integrating rational polymer design and minimally invasive microneedle delivery, MPEG‑PCL@ME MNs effectively enhance the optical stability of ME, improve lymphatic targeting and imaging quality, and exhibit favourable biosafety, thereby representing a promising platform for high‑quality lymphography and functional lymphatic assessment.

## Conclusions

This work proposes a straightforward and noninvasive method based on a novel lymphatic tracer (MPEG-PCL@ME) that can be administered intradermally via microneedles and utilizing imaging via a portable detection device to visualize and quantify the lymphatic system. Compared with the ME solution, the MPEG-PCL@ME solution has better targeting capabilities, stability, optical qualities, and biocompatibility. Moreover, in contrast to ME, imaging with MPEG-PCL@ME *in vivo* clearly demonstrated the morphology of lymphatic vessels and nodes with higher imaging quality in the rat tail. In addition, only the detailed lymphatic structures and functions, including those of the dominant lymphatic vessels and the lymphatic pump, can be detected with the MPEG-PCL@ME application. As a result, MPEG-PCL@ME can be utilized as a lymphatic tracer to assess the lymphatic system’s structure and function, while microneedles can deliver the tracer in a more convenient and painless manner, satisfying the growing demand for lymphangiographic evaluation and early disease diagnosis. Although our research has several important implications, certain limitations remain. Future research should expand the application of this novel lymphatic tracer to cancer development and metastasis and impaired wound healing.

## Supplementary Material

Supplementary_File_tkaf067

## Data Availability

The data that support the findings of this study are available from the corresponding author upon reasonable request.
