# Supplementary material for: A novel method for targeting lymphatic vessel imaging: methylene blue nanoparticle integrated with dissolvable microneedles
Source: Burns Trauma. 2026 Jan 28;14:tkaf067. doi: 10.1093/burnst/tkaf067 (PMC13340466; doi:10.1093/burnst/tkaf067)
Supplement: Supplementary_File_tkaf067 [file supplementary_file_tkaf067.docx]

**Supporting information**

**A novel method for targeting** **lymphatic vessel** **imaging: methylene blue nanoparticle integrated with dissolvable microneedles**

*Chengyao Han^1+^, Beibei Wu^2+^, Chunxiao Cui^3+^, Peiru Min^1^，Xinxian Meng^1^, Yuhao Sun^1^, Ke Wen^1^, Chuanliang Feng^2^**, Yixin Zhang^1^*, Xueqian Wang^1^*, Ke Li^1^**

^1^ Department of Plastic and Reconstructive Surgery, Shanghai Ninth People’s Hospital, Shanghai Jiao Tong University School of Medicine, Shanghai 200011, China

^2^ State Key Lab of Metal Matrix Composites, Shanghai Key Laboratory for Molecular Engineering of Chiral Drugs, School of Materials Science and Engineering, Shanghai Jiao Tong University, Shanghai 200240, China

^3^ Department of Burns and Plastic Surgery, Shanghai Children’s Medical Center, Shanghai Jiao Tong University, Shanghai 200127, China

* Corresponding authors: [18817821624@163.com](mailto:18817821624@163.com) (K. Li); [wxq0729@sjtu.edu.cn](mailto:wxq0729@sjtu.edu.cn) (X. Q. Wang); [zhangyixin6688@hotmail.com](mailto:zhangyixin6688@hotmail.com) (Y. X. Zhang)

*^+^* These authors equally contributed to this work.

**1. Experimental section**

**1.1. Materials**

ɛ-CL (ε-caprolactone), MPEG (methoxy poly(ethylene glycol)), ME (methylene blue), PVA (polyvinyl alcohol), stannous caprylate, toluene, dichloromethane, ether, and HA (hyaluronic acid) were purchased from Aladdin Chemistry (Shanghai) Co., Ltd.; Mouse lymphatic endothelial cells (LECs) was purchased from Procell Life Science & Technology (Wuhan) Co., Ltd.; Endothelial cell growth medium (PromoCell), FBS (fetal bovine serum), PS (penicillin-streptomycin), 0.25% trypsin, PBS (phosphate-buffered saline), 5% glucose, and Triton X-100 were purchased from Thermo Fisher Scientific (China) Co., Ltd.; AM/PI (Calcein/propidium iodide), F-actin Staining Kit, and DAPI (4',6-diamidino-2-phenylindole) were bought from Shanghai Shaoxin Biotechnology Co., Ltd.. All aqueous solutions were prepared using ultrapure water (18 MU) from a Milli-Q system (Millipore) and all of the chemicals were used as received without further purification.

**1.2. Release study of** **MPEG-PCL@ME MNs in vitro**

The release study was performed using a vertical Franz diffusion cell system. The cumulative amount of drug release was calculated following the Equation (1):

*Q_n_ (%)* = $\frac{VC_{n}+ \sum_{i=1}^{n-1} C_{i}V_{i}}{A}$ × *100* (1)

where *Q_n_* is the cumulative percentage of MPEG-PCL@ME permeated across the skin, *V* is the volume of the medium in the acceptor chamber (7 mL), *V_i_* is the volume of the medium sampled at various time points (1 mL), *C_n_* is the concentration of MPEG-PCL@ME in the medium in the acceptor chamber at various time points, *C_i_* is the concentration of MPEG-PCL@ME in the medium in the acceptor chamber at the *i*th (n − 1) time point, and A is the feeding quantity of the MPEG-PCL@ME.

**1.3. Preparation and Characterization of MPEG-PCL**

The dried MPEG and ε-CL were placed in a three-port round-bottom flask with stannous caprylate as catalyst and toluene as reaction solvent. The reaction was repeatedly vacuumed and filled with dry nitrogen for at least 3 times. MPEG-PCL was synthesized by ring-opening polymerization under magnetic stirring in oil bath at 90℃. After 24 hours of reaction, the oil bath was switched off, and nitrogen was turned off once the reaction system had cooled to room temperature, obtaining the crude MPEG-PCL. To remove the remaining toluene, the product was rotated under reduced pressure at 90℃ for 2 hours. After cooling to room temperature, a certain amount of dichloromethane was added to dissolve the reaction product completely. It was then precipitated with ether, stood at 4℃, and pumped and filtered under reduced pressure to obtain a white precipitate. The process was repeated three times, and finally bottled and sealed, and stored at -20℃. The chemical structure and molecular weight of MPEG-PCL were analyzed by NMR spectrometer (600-MHz Bruker) and Gel permeation chromatography system (GPC, LC-20AD XR), respectively.

**1.4. Hemolysis testing**

The percent hemolysis was calculated by measuring the absorbance of the supernatant solution at 545 nm using a UV-Vis Spectrophotometer (Evolution 201). The hemolysis ratio was calculated by Equation (2):

Hemolysis ratio =(*OD sample* – *OD negative*) / (*OD postive* - *OD negative* ) (2)

where *OD sample* is the absorbance of samples at 545 nm, *OD negative* is the absorbance of normal saline at 545 nm, and *OD positive* is the absorbance of TrionX-100 at 545 nm.

**1.5. Lymphatic transport experiment**

MPEG-PCL@ME transport across LECs was assessed by using a transendothelial transport model in vitro. Briefly, LECs were grown in endothelial cell growth medium for 48 hours on the bottom of transwell inserts. After that, the apical side of LECs was treated with MPEG-PCL@ME, and samples were taken from the basolateral compartment every 2 hours for up to 24 hours. Effective permeability was estimated by Equation (3):

*Transported (normalized)* = *C_bottom_V_bottom_* / *tSC_initial_* (3)

where *C_initial_* is the concentration of MPEG-PCL@ME, *V_bottom_* is the volume of the basolateral compartment, S is the surface area, and t is the preset time.

**2. Additional Experimental Data and Figures**

**
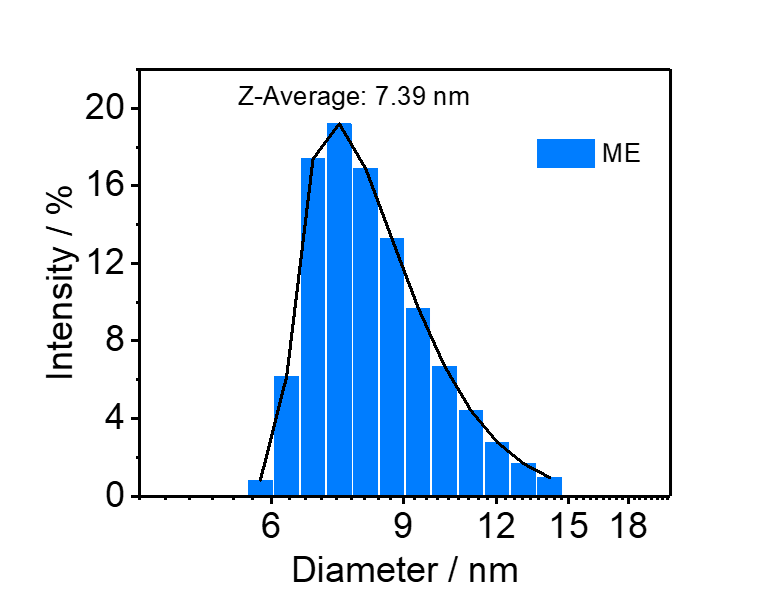
**

Figure S1. Size distribution of ME.


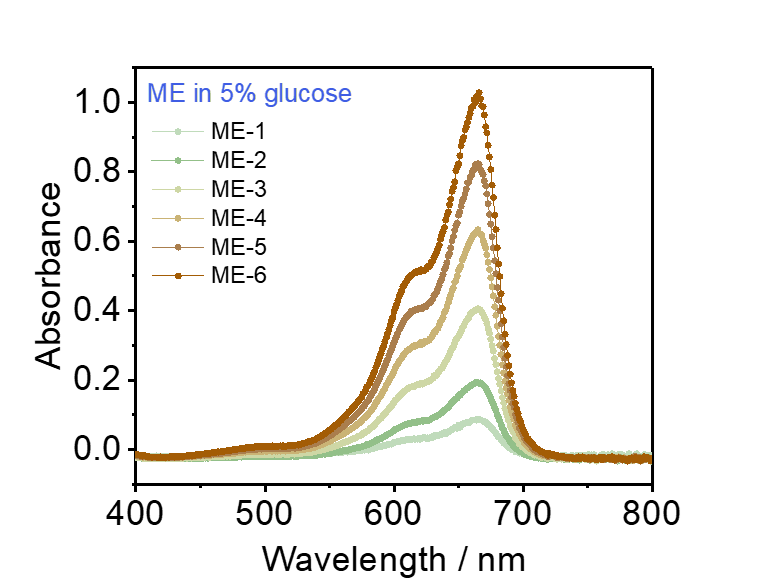


Figure S2. Absorption spectra of MB at various concentrations in 5% glucose solution.


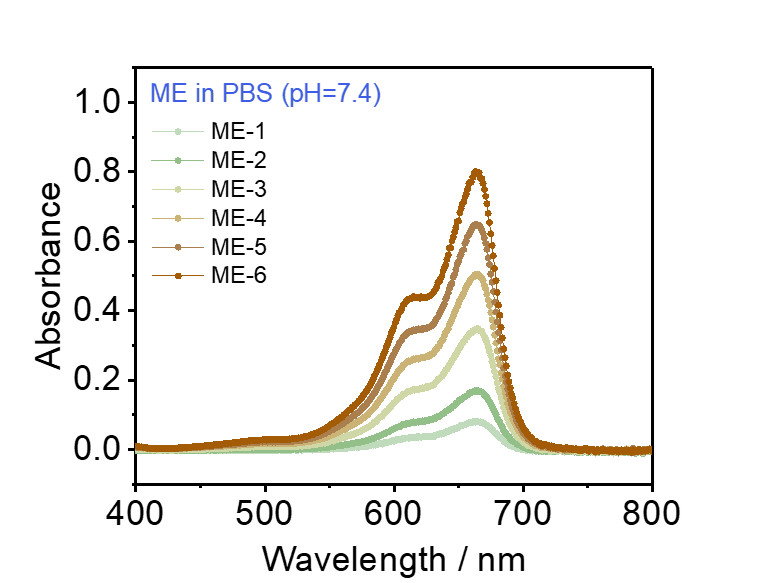


Figure S3. Absorption spectra of MB at various concentrations in PBS solution.


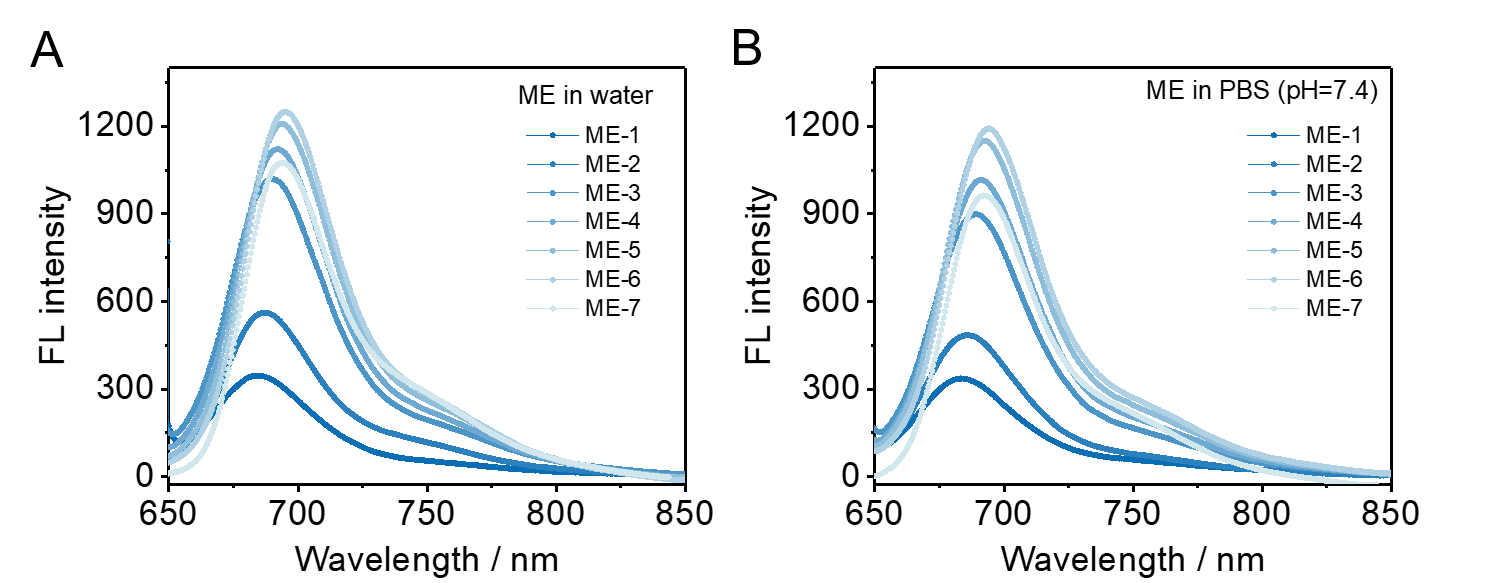


Figure S4. Fluorescence spectra of MB with different concentrations in water and PBS (pH=7.4) solution.


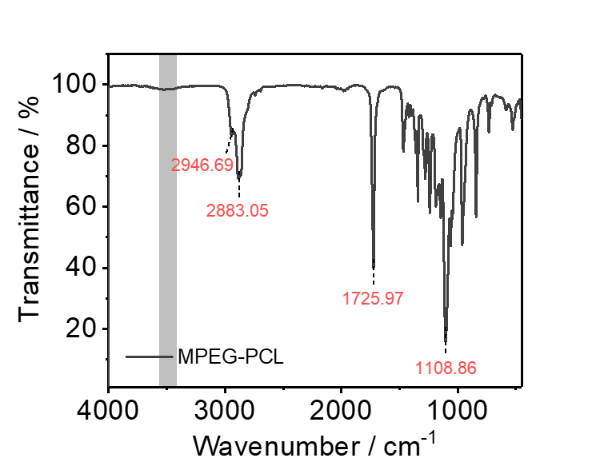


Figure S5. FTIR spectrum of MPEG-PCL.


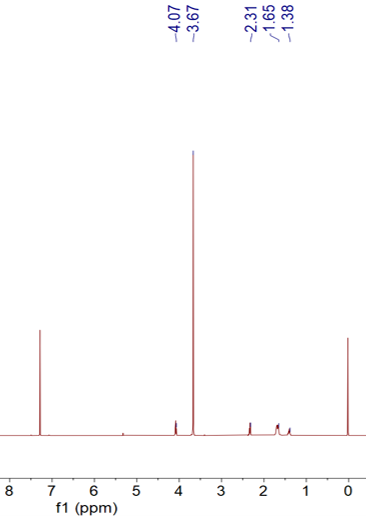


Figure S6. ^1^H-NMR spectrum of MPEG-PCL in DMSO-d6.


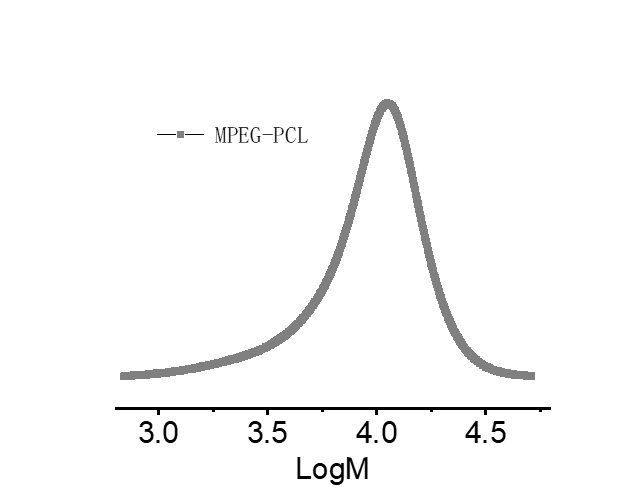


Figure S7. GPC chromatogram of the MPEG-PCL.


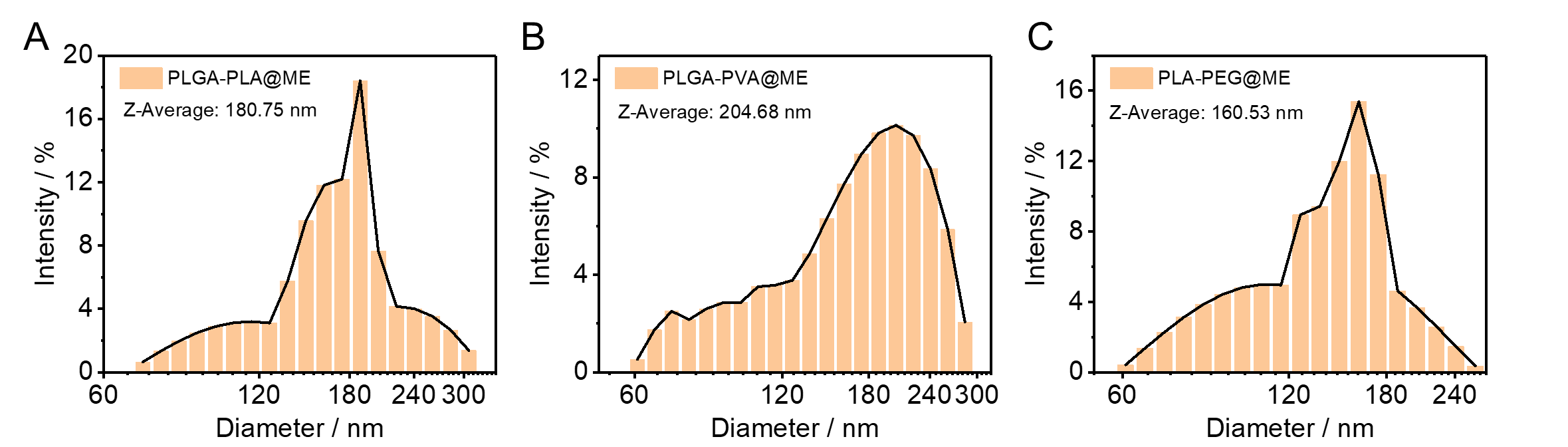


Figure S8. Size distribution of (A) PLGA-PLA@ME, (B) PLGA-PVA@ME, and (C) PLA-PEG@ME.


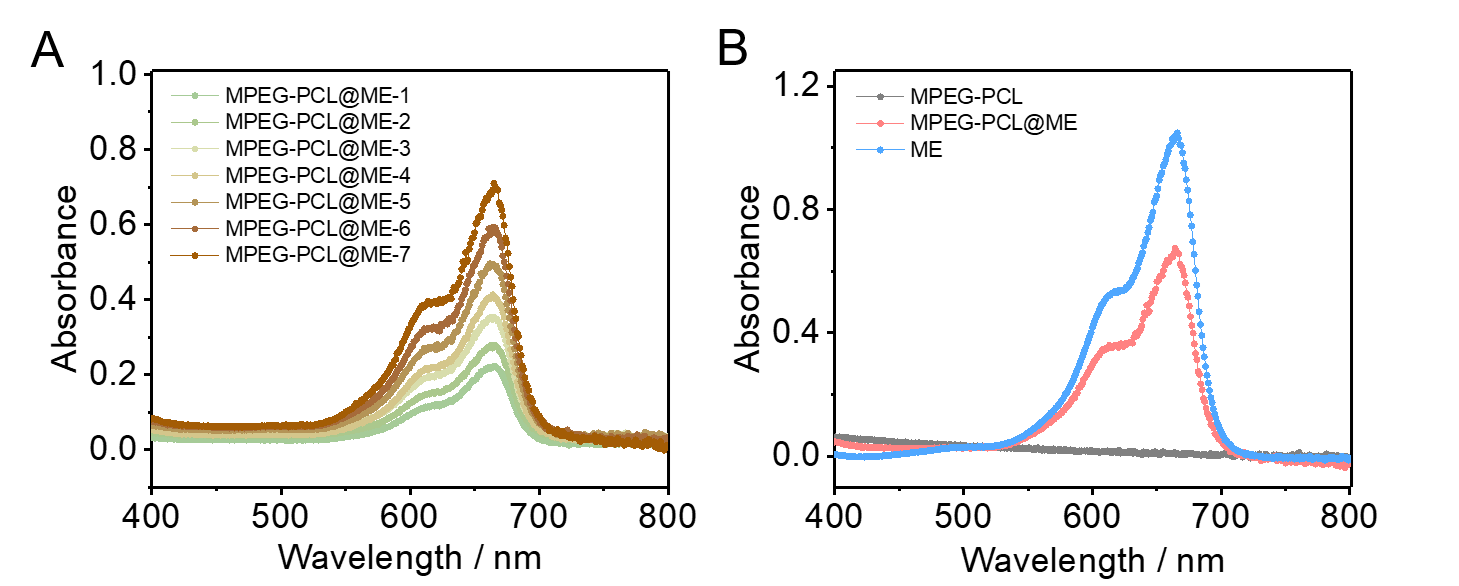


Figure S9. Absorption spectra of (A) MPEG-PCL@ME at various concentrations and (B) MPEG-PCL, MPEG-PCL@ME, and ME.


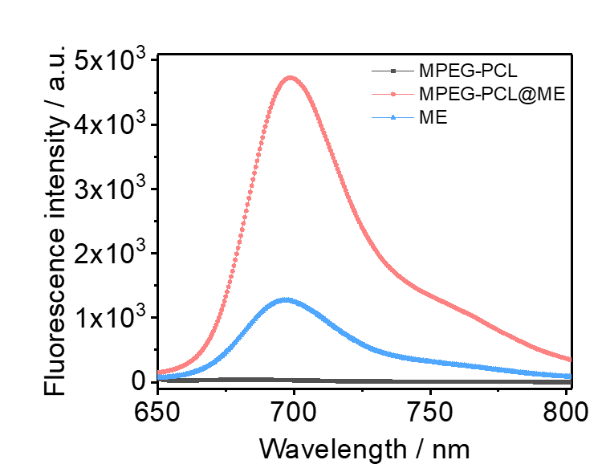


Figure S10. Fluorescence spectra of MPEG-PCL, MPEG-PCL@ME, and ME.


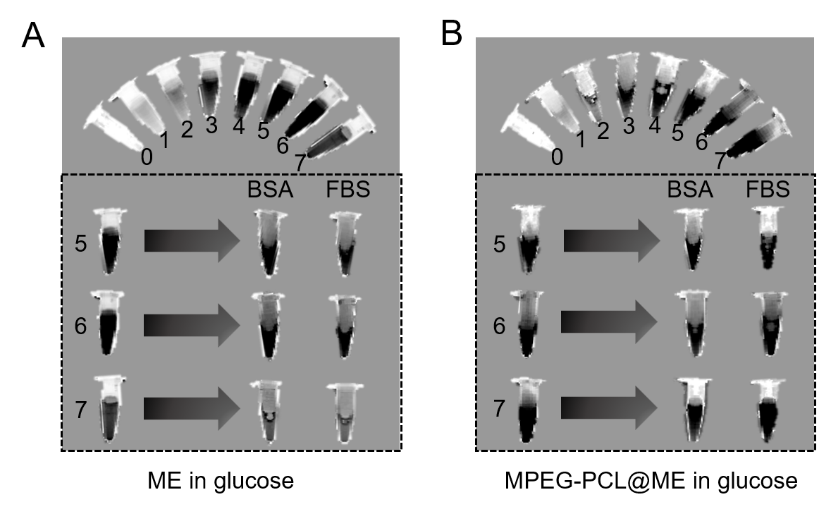


Figure S11. Fluorescence images of ME and MPEG-PCL@ME in glucose at various concentrations.


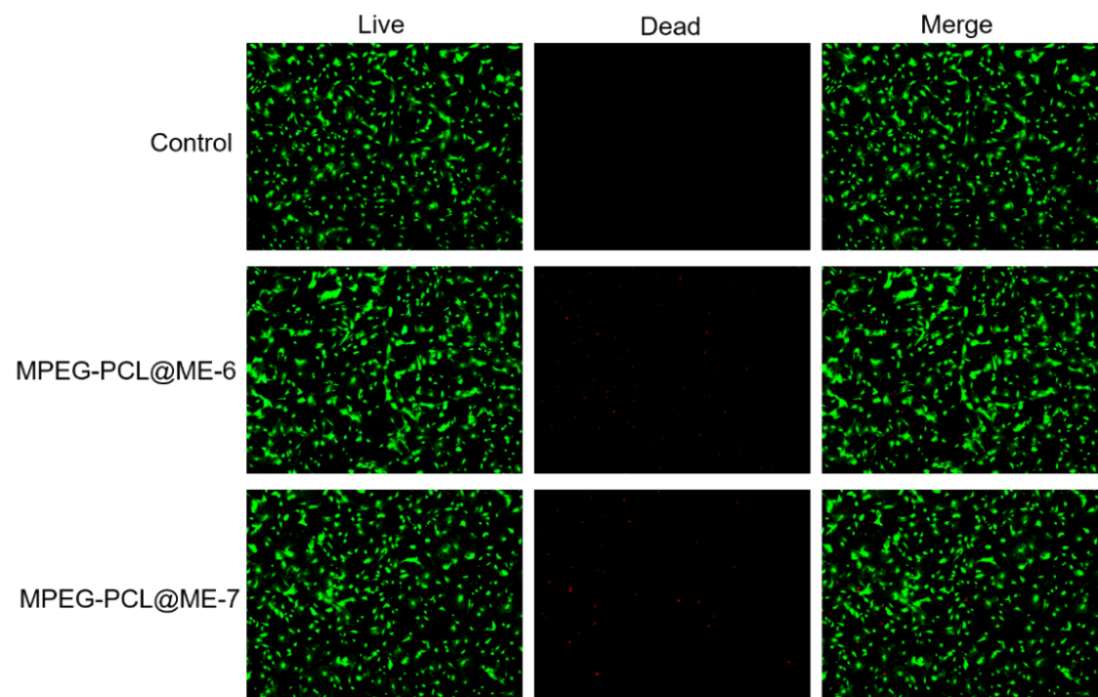


Figure S12. Fluorescence images of the live/dead experiment of MPEG-PCL@ME at various concentrations. Scale bar, 200 μm.


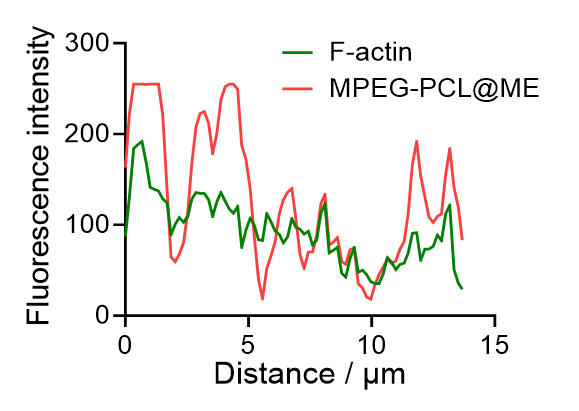


Figure S13. MPEG-PCL@ME fluorescence co-localizes with F-actin fluorescence.


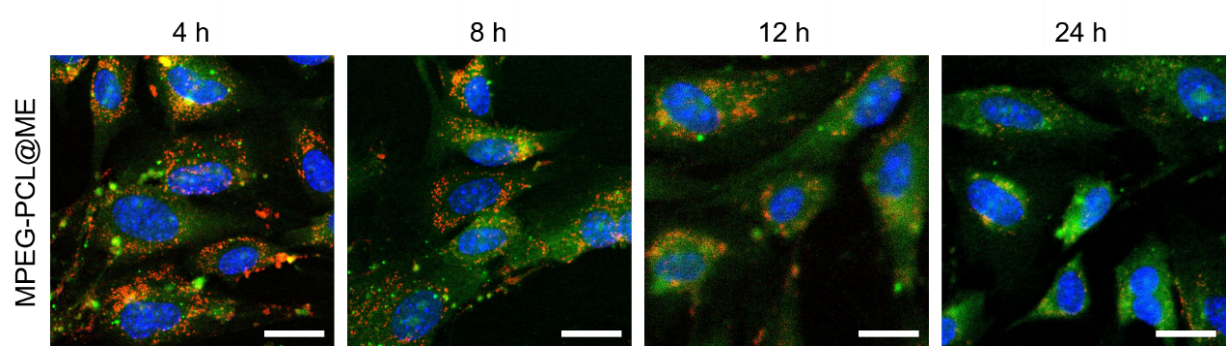


Figure S14. Confocal fluorescence image of LECs stained for F-actin (green) and DAPI (blue) treated with MPEG-PCL@ME (red) at various times. Scale bar, 25 μm.


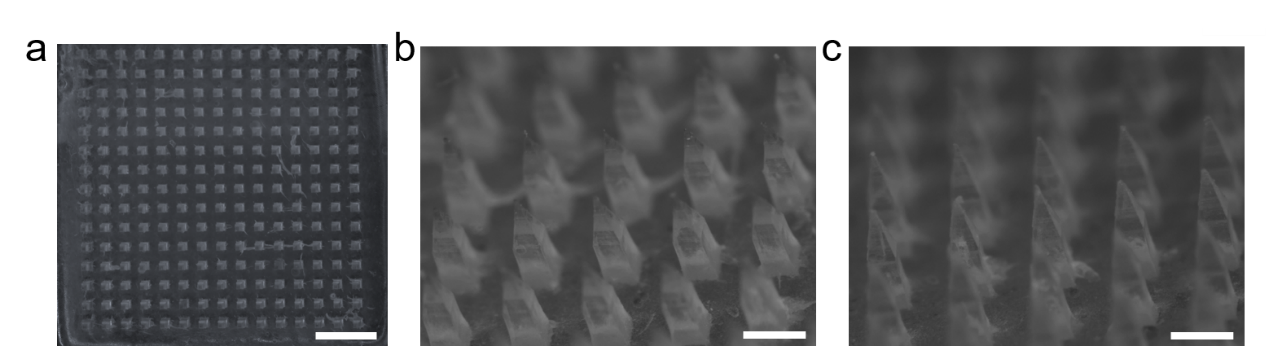


Figure S15. Stereomicrographs of MPEG-PCL@ME MNs. Scale bar, 2 mm in (a) and 500 μm in (b, c).


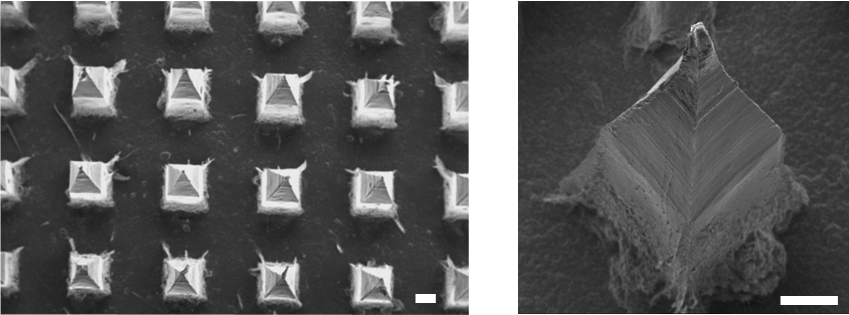


Figure S16. SEM images of MPEG-PCL@ME MNs. Scale bar, 100 μm.


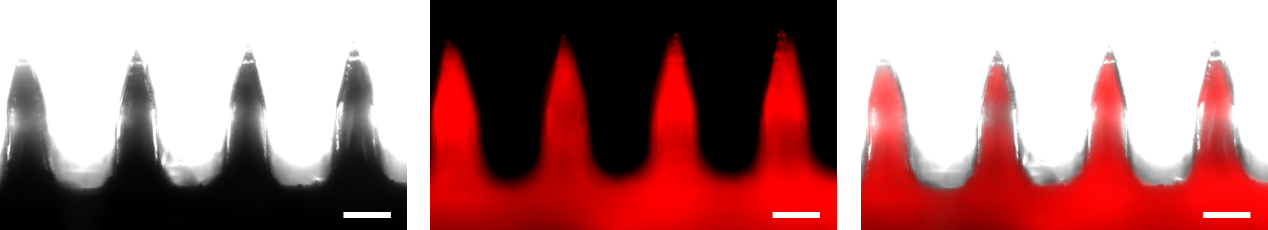


Figure S17. Fluorescence images of MPEG-PCL@ME MNs. Scale bar, 300 μm.


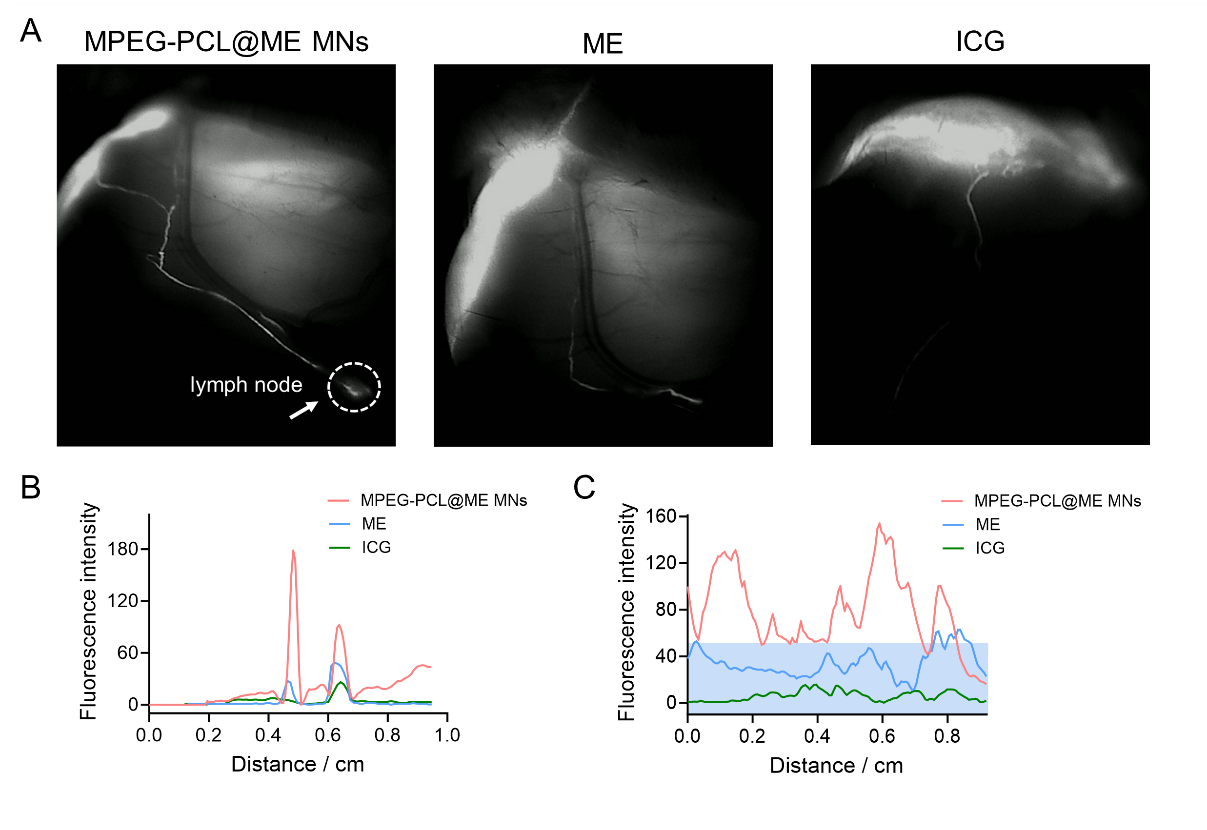


Figure S18. (A) Fluorescence imaging of normal lymphatic vessels on one side of the rat hind limb after treatment with MPEG-PCL@ME MNs and injection of ME and ICG, respectively. Quantitative analysis of fluorescence intensity of (B) the cross-sectional level and (C) the longitudinal axis of rat hind limb lymphatic vessels of MPEG-PCL@ME MNs, ME, and ICG.
